# Green Revolution to Gene Revolution: Technological Advances in Agriculture to Feed the World

**DOI:** 10.3390/plants11101297

**Published:** 2022-05-12

**Authors:** Mohd Fadhli Hamdan, Siti Nurfadhlina Mohd Noor, Nazrin Abd-Aziz, Teen-Lee Pua, Boon Chin Tan

**Affiliations:** 1 Centre for Research in Biotechnology for Agriculture, Universiti Malaya, Kuala Lumpur 50603, Malaysia; fadhlihamdan@um.edu.my; 2 Institute of Microengineering and Nanoelectronics (IMEN), Universiti Kebangsaan Malaysia, Bangi 43600, Malaysia; sitinurfadhlina@ukm.edu.my; 3 Innovation Centre in Agritechnology for Advanced Bioprocessing (ICA), Universiti Teknologi Malaysia, Pagoh 84600, Malaysia; nazrin.abdaziz@utm.my; 4 Topplant Laboratories Sdn. Bhd., Jalan Ulu Beranang, Negeri Sembilan 71750, Malaysia; teenleepua@um.edu.my

**Keywords:** biotechnology, breeding, crop improvement, genetically modified crops, genome editing

## Abstract

Technological applications in agriculture have evolved substantially to increase crop yields and quality to meet global food demand. Conventional techniques, such as seed saving, selective breeding, and mutation breeding (variation breeding), have dramatically increased crop production, especially during the ‘Green Revolution’ in the 1990s. However, newer issues, such as limited arable lands, climate change, and ever-increasing food demand, pose challenges to agricultural production and threaten food security. In the following ‘Gene Revolution’ era, rapid innovations in the biotechnology field provide alternative strategies to further improve crop yield, quality, and resilience towards biotic and abiotic stresses. These innovations include the introduction of DNA recombinant technology and applications of genome editing techniques, such as transcription activator-like effector (TALEN), zinc-finger nucleases (ZFN), and clustered regularly interspaced short palindromic repeats/CRISPR associated (CRISPR/Cas) systems. However, the acceptance and future of these modern tools rely on the regulatory frameworks governing their development and production in various countries. Herein, we examine the evolution of technological applications in agriculture, focusing on the motivations for their introduction, technical challenges, possible benefits and concerns, and regulatory frameworks governing genetically engineered product development and production.

## 1. Introduction

**Figure 1:**
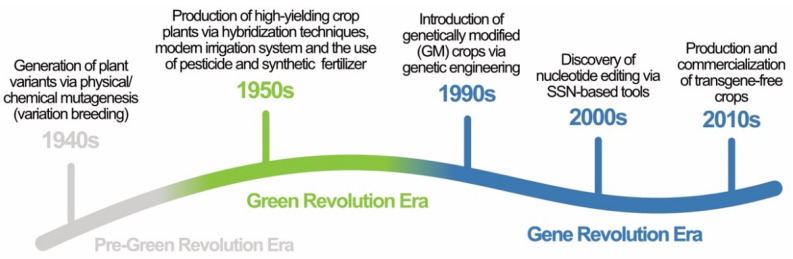
A roadmap showing the shift from the Green Revolution era to the Gene Revolution era. The Pre-Green Revolution, Green Revolution, and Gene Revolution eras are marked in grey, green, and blue, respectively. The important events and years are mentioned in their corresponding eras.

Agriculture plays a critical role in transforming economies and promoting food security [1]. Apart from providing livelihoods for 570 million farmers worldwide [2], the current global agricultural output feeds 7.5 billion people, with three major crops, rice (*Oryza sativa*), corn (*Zea mays*), and wheat (*Triticum aestivum*), providing over 60% of our food energy intake [3]. To keep up with the food demand, technological applications in agriculture have evolved substantially to maximize growth yield. Early innovation, such as the conventional selective breeding technique, involves crossing desired parental plants and selecting offspring with relevant combined characteristics [4]. This technique resulted in high-yielding plant varieties in wheat and rice during the ‘Green Revolution’ era in the 1950s (Figure 1). Indeed, selective breeding techniques and other improvements in agricultural practices (e.g., improved irrigation systems, chemical fertilizers, and heavy machinery) have tremendously increased global food production over the past few decades [5,6,7]. However, as food demand is expected to increase by 60% [8] to feed 9.7 billion people in 2050 [9], a novel strategy is needed to promote food security [1].

Conventional breeding techniques have their disadvantages, such as (1) uncontrolled genetic mutations, (2) the need for the parental plants to be closely related to produce offspring, and (3) the laborious and time-consuming procedure of crossing and backcrossing hybrids to obtain the elite lines [5,10]. Unlike conventional methods, modern biotechnology tools provide a more specific and controlled way of altering plant DNA or proteins. This may result in high-yielding and nutritious crops and/or those which are more resilient to extreme weather, arid conditions, and diseases [11]. As biotechnology tools overcome the limitations associated with conventional techniques, they are considered promising tools to further improve global agronomic output and promote food security. However, concerns regarding biotechnology applications have been raised by critics. They include biosafety issues, ethical issues, and the long-term impact on human health and the environment. 

In this review, we describe technological progress in crop improvement, focusing on the shift from the use of conventional breeding techniques during the ‘Green Revolution’ era towards the more complex molecular techniques in the so-called ‘Gene Revolution’ era (Figure 1). The motivation behind this shift is examined. We then identify the distinction between several modern biotechnology tools and highlight their definitional ambiguities in the current regulatory framework. We also discuss the current regulatory frameworks governing genetically modified (GM) food production in certain countries and suggest how the newer gene-editing tools should fit into these regulatory frameworks. The current benefits and concerns associated with biotechnology-derived foods are also listed and discussed. Ultimately, the review addresses how modern biotechnology tools complement traditional methods by offering novel ways to improve crops and food production. 

## 2. Mutation Breeding to Increase Plant Varieties

Scientists have long acknowledged the role of naturally occurring mutations in plants that may produce traits that thrive in the changing environment. However, since natural mutations occur by chance, the possibility of superior traits emerging naturally happens very slowly [12]. Thus, ‘mutation breeding’ was developed to induce random mutations to mimic and expedite this process. Pioneering studies on radiation-induced mutations using X-rays on Drosophila [13], and plants, such as barley [14] and corn [15], laid the foundation for gene function and repair studies. These studies paved the way for mutational breeding applications in agriculture [16]. Later, the use of highly energetic gamma-rays further accelerated the mutational breeding process, in which the number of mutations occurring per cell was affected by different levels of radiation imposed on the plants, including the distances of the plant to the radiation source or the exposure time [17]. Chemical-induced mutagenesis of plants was achieved using alkylating agents, such as ethylmethanesulfonate (EMS), methylnitrosourea (MNU), and ethylnitrosourea (ENU). Among these alkylating agents, EMS has been widely used to introduce random point mutations in plants to generate variants of agronomic importance [18].

In the recent decade, fast neutron radiation-induced mutagenesis has been increasingly used due to its efficiency in rapidly generating a library of knockout genes [19]. Its ability to create multiple deletions ranging from 1 bp to 18 Mb has been valuable in delineating several biosynthesis pathways in important crops, such as raffinose family oligosaccharide synthesis [20] and seed protein synthesis in the soybean (*Glycine max*) [21]. To date, there are about 3200 radiation-induced plant varieties produced from 210 plant species cultivated in over 70 different countries [16]. However, undirected mutagenesis techniques have their own disadvantages, including: (1) the ‘randomness’ of the mutation induction, which may cause off-target alterations across the plant genome; (2) limitations in producing desirable dominant alleles; and (3) the laborious work of screening out mutant plants with desirable traits from a huge population [22]. Therefore, a more direct and efficient way of DNA alteration in the plant genome is desirable to accelerate the production of desirable variants while reducing the risk of off-target mutations.

## 3. Breeding Strategies to Increase Crop Yield

Plant hybridization is a widely used conventional technique to produce crops with better characteristics, such as better yield, improved color, and increased resilience to environmental stresses and diseases [23]. It involves crossing two different species or varieties of the same plant by transferring the pollen of a plant into the female part of a different plant resulting in ‘hybrids’. This gene method is different from another selective breeding technique referred to as ‘inbreeding’ that involves two genetically similar or biologically related parents. The main goal of hybridization is to acquire the best possible characteristic(s), while inbreeding aims to maintain stable characteristic(s) over time [24,25]. 

Since most crops are hermaphroditic (having both male and female organs on the same flower), the hybridization technique always seeks to reduce or avoid the process of self-pollination during flowering [26]. One way to achieve this is to make the female line male-sterile via mechanical or genetic alteration [27]. One prominent example of the former is the detasseling (the process of removing the tassel) of maize which is a cost- and time-productive method due to the presence of the anthers at the top of the plant [28]. However, mechanical sterilization is not commercially applicable for other crops that consist of the male and female components coexisting in the same flower [27]. Therefore, a genetic means was used to avoid self-pollination using self-incompatibility (SI) or cytoplasmic male sterility (CMS) systems [29]. 

The SI system largely depends on the S-locus, where tightly linked genes form co-adapted haplotypic combinations which control pollen and pistil specificities. After pollination, the pollen will be rejected if the specificity is encoded by the same haplotype as that of the pistil [30]. In CMS, the production of functioning pollen is prohibited by virtue of mitochondrial dysfunction, which produces open reading frames (ORFs) that induce pollen disruption. Spontaneous fertility reversion is possible, although happening at a relatively low rate in major crops, such as rice and corn, and other crops, such as carrot, common bean, and mustard greens [31]. Both SI and CMS systems are still used today. However, they are limited to the sporophytic system, such as *Brassica*, and gametophytic systems in Papaveraceae, Solanaceae, Rosaceae, and Plantaginaceae [32]. The difficulties in developing and applying these systems, however, hinder their utilization for other crops.

## 4. The Rise of Recombinant DNA Technology and Genetically Modified (GM) Crops

The next major technological landmark in agriculture involves recombinant DNA technology (also known as ‘genetic engineering’ or ‘genetically modified organism [GMO] technology’). This technology was used to produce mostly herbicide- or pesticide-resistant GM crops in the early 1990s, marking the start of the ‘Gene Revolution’ era [33]. The inception of DNA recombination technology started earlier with the exploitation of *Agrobacterium tumefaciens* to transfer transgenes into host plants (Table 1). This eventually opened a new realm in crop improvement [34,35]. Bevan et al. [36] demonstrated that a chimeric antibiotic-resistant gene could be transferred into tobacco (*Nicotiana tabacum*), and the transformed plant cells could be selected on antibiotic-supplemented growth media. The first virus-resistant transgenic plant expressing tobacco mosaic virus (TMV) coat protein was then generated, showing delayed symptom development when infected with TMV compared to non-transgenic lines [37]. The first transgenic insect-resistant plant was generated in the following year by expressing an insecticidal Bt2 protein from *Bacillus thuringiensis* (Bt) in tobacco [38]. 

Biotechnology has opened up a new research frontier using RNA interference (RNAi) with the discovery of the ‘co-suppression’ phenomenon that caused gene silencing in petunia (*Petunia hybrida*) [45]. Unexpectedly, an attempt to overexpress chalcone synthase (CHS), a key enzyme in anthocyanin biosynthesis, in petunia did not produce flower petals with darker pigment [45]. Instead, the integration of the chimeric petunia *CHS* transgene resulted in either white or partially white flower petals in 42% of the transgenic petunia. This indicated a possible ‘co-suppression’ phenomenon between the endogenous and the introduced CHS genes, which caused a block in the anthocyanin biosynthesis [45]. The ‘co-suppression’ mechanism was further elucidated in subsequent work by Lindbo et al. [59] on tobacco etch virus (TEV)-resistance in transgenic tobacco. In their experiment, the transgene mRNA was abundantly detected in the noninfected plants while barely detected in the transgenic tobacco that recovered from the TEV infection. This strongly suggested the co-suppression of the virus and that the transgene must operate at the RNA level since the TEV has an RNA genome. 

The simplicity of the RNAi mechanism makes it an effective tool for gene knockdown studies. This eventually led to the production of transgenic crops with commercial value. A prominent example is the commercialization of the ‘Flavr Savr’ tomato (cherry tomato; *Lycopersicon esculentum*) in 1993, approved by the US Food and Drug Administration (FDA) (Table 1). The Flavr Savr tomato was based on RNAi technology, where an antisense expression cassette of the endogenous polygalacturonase (PG) gene was integrated into the tomato. PG was found to dissolve cell-wall pectin in the fruit ripening process. By silencing the PG expression, the transgenic Flavr Savr tomato had a longer shelf-life than the non-transgenic tomato [60]. In 1995, the US Environmental Protection Agency (EPA) approved the first pesticide-producing food crops (Bt potato and Bt corn) and non-food crops (Bt cotton) [49]. In the following year, transgenic glyphosate-resistant soybean was the first herbicide-resistant crop to be marketed for the consumer market in the USA [50].

The adoption of GM crops has been swift in the USA. The cultivation area of GM crops in the USA has increased from 3 million hectares (ha) in 1996 [61] to 71.5 million ha in 2019 [62]. Similarly, the global adoption of GM crops has substantially increased since their first introduction in 1996. The global cultivation area has increased 112-fold from only 1.7 million ha in 1996 to 190.4 million ha in 2019 [62]. A total of 44.2%, 43.9%, 9.9%, and 1.5% of GM crops planted in the world can be found in North America, South America, Asia, and Africa, respectively. Only 0.32% and 0.05% of the GM crops were planted in Oceania and Europe, respectively [62]. The top producer of GM crops in 2019 was the USA (71.5 million ha), followed by Brazil (52.8 million ha) and Argentina (24.0 million ha). Up to 17 million farmers in 29 countries have planted 14 types of GM crops with various traits of improvement, including soybean, corn, cotton, canola (*Brassica napus*), alfalfa (*Medicago sativa*), sugar beet (*Beta vulgaris*), sugarcane (*Saccharum officinarum*), papaya (*Carica papaya*), safflower (*Carthamus tinctorius*), potato (*Solanum tuberosum*), eggplant (*Solanum melongena*), squash (*Cucurbita*), apple (*Malus pumila*), and pineapple (*Ananas comosus*) [62]. 

**Figure 2:**
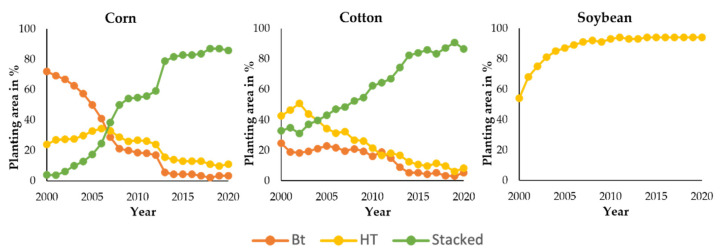
The percentage of genetically modified (GM) trait varieties of all planting areas for corn, cotton, and soybean in the USA from 2000 until 2020. Insect resistance (Bt), herbicide resistance (HT), and stacked-gene (Stacked) varieties are indicated by orange, yellow, and green colors, respectively. Figure assembled using the annual GE crop adoption data from the US Department of Agriculture (USDA)’s Agricultural Marketing Service website.

**Figure 3:**
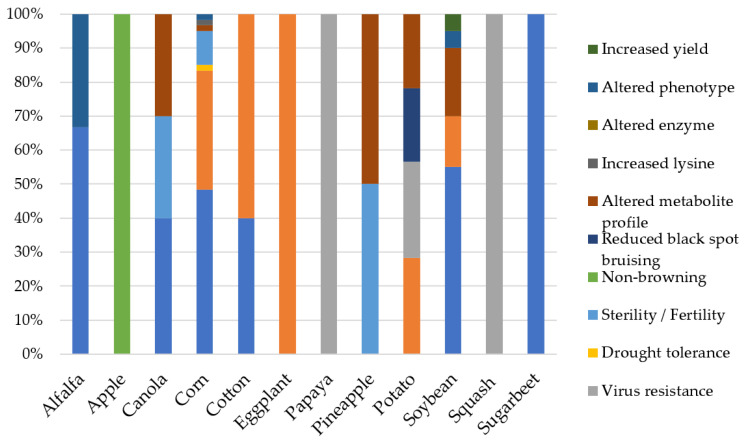
Traits selected for improvement in GM crops. The list of traits (indicated by different colors) was obtained from USDA’s Agricultural Marketing Service website, which maintains a list of bioengineered food.

The first generation of GM crops contained only a single introduced trait (mono-trait) [63]. Adding new transgenes to an existing transgenic plant has been challenging due to difficulties in introgressing additional transgenic traits that segregate independently [64]. As recombinant DNA technology advances, multiple traits can now be integrated within the same GM plant, a process known as ‘gene stacking’ [64]. This innovation has become the preferred strategy in GM crop production, especially in the USA. In 2020, the cultivation of gene-stacked GM corn and GM cotton (*Gossypium*) covered 86% and 87% of their total cultivation areas, compared to 4% and 33% in 2000, respectively (Figure 2). Insect tolerance, herbicide tolerance, and virus resistance are the three most introduced individual traits in GM crops (Figure 3). 

In recent years, several newer approaches to facilitate gene stacking in crops have been reported, such as marker-assisted selection (MAS)-based gene pyramiding [65] and gene assembly in *Agrobacterium* by nucleic acid transfer using recombinase technology (GAANTRY) [66]. Newer genome editing tools that allow repeated gene integration within the same transgenic loci will further expedite the production of stacked-gene varieties. 

Climate change has further increased the need to produce improved crops with better resilience in the ever-changing environment. If the issue is not addressed, climate change may soon disrupt food availability, reduce access to food, and affect food quality and yield [67,68,69,70]. Therefore, continuous innovations require to be made to enable specific changes in the crop genome to speed up the process of identifying plants with desirable traits. 

## 5. A New Era of Genome Editing Using Sequence-Specific Nuclease (SSN)-Based Tools

The first generation of GM plants usually involves the random insertion of foreign genes throughout the host plant genome. Unlike GM technology, newer genome editing tools use sequence-specific nucleases (SSN) which allow alteration of pre-determined DNA sequences in the host plant genome by harnessing native DNA repair machinery [71]. Due to their specificity, SSN-based tools have been widely applied to introduce compositional changes or to produce novel variants with higher resilience against biotic and abiotic stresses [72]. To date, there are four major SSNs that have been used in genome editing, including engineered homing endonucleases or meganucleases, zinc finger nucleases (ZFNs), transcription activator-like effector nucleases (TALENs) and clustered regularly interspersed short palindromic repeat (CRISPR)/Cas9 systems [73,74,75,76].

**Figure 4:**
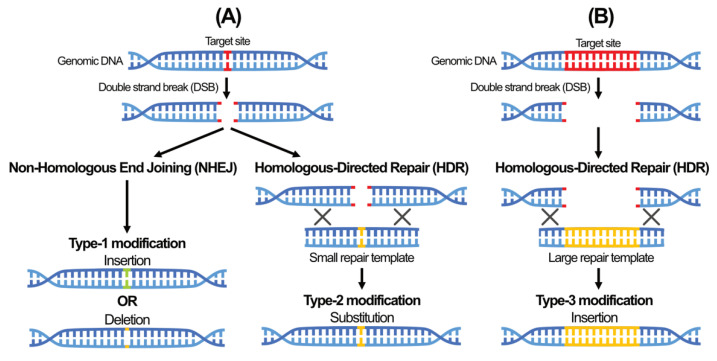
Three types of modifications are mediated by sequence-specific nuclease (SSN) tools in genome editing. (**A**) Small site alteration on the genomic DNA can be made via the non-homologous end joining (NHEJ) or homologous-directed repair (HDR) pathway. Without a repair template, the error-prone NHEJ may result in Type-1 modification (small insertion and/or deletion). With a small repair template, Type-2 modification (small substitution) can be made via the HDR pathway. (**B**) Large site alteration with a longer repair template will initiate the HDR-mediated, Type-3 modification (large DNA sequence or transgene integration). Red bars indicate the target site where the double-strand break is made on the genomic DNA. Yellow bars indicate new DNA sequences from the repair template that are integrated into the target site via the HDR pathway. The green bar indicates a single nucleotide inserted in a template-independent manner via the NHEJ pathway.

SSN acts as a molecular ‘scissor’ to induce double-strand breaks (DSB) at or near the site of interest in the plant genome [77]. This triggers either the error-prone non-homologous end-joining (NHEJ) mechanisms that delete a single, or set of, DNA sequence(s) at the repair site or homologous-directed repair (HDR), which allows single base substitution or whole gene replacement [78] (Figure 4). The resulting DNA modification can be classified as Type-1 (small insertion/deletion), Type-2 (substitution), or Type-3 (large insertion), depending on the repair mechanism pathway initiated [79]. 

In Type-1 modification, the cell’s native repair machinery re-joins the DNA breakage via the NHEJ mechanism (Figure 4). The mutated sequence can then be isolated by self- or back-crossing to remove all the transgenes expressing the SSN machinery from the resulting mutants. The simplicity of the CRISPR/Cas9 system for producing Type-1 modifications has made it the most used approach for crop improvement compared to the other SSNs [80]. In addition, recent loss-of-function (knockout) studies using SSN-based techniques have elucidated various gene functions during plant developmental stages. For example, transformed potatoes showed increased late blight resistance when susceptibility (S) genes, namely *StDND1*, *StCHL1*, and *StDMR6-1*, were knocked out using CRISPR/Cas9-based editing [81]. Resistance against bacterial blight was also improved in rice by editing *OsSWEET11*, *OsSWEET13*, and *OsSWEET14* genes via CRISP/Cas9 [82]. In addition, in rice, resistance against the seed-borne rice pathogen, *Burkholderia glumae*, was improved with CRISPR/Cas9-based mutation in *Oryza sativa MITOGEN-ACTIVATED PROTEIN KINASE 5* (*OsMPK5*) [83]. In another study, enhanced rice grain size and yield were observed by editing several genes (*Gn1a*, *DEP1*, *GS3*, and *IPA1*) that are responsible for the plant architecture [84]. CRISPR/Cas-targeted mutation in tomato (*Solanum lycopersicum*), *SlJAZ2*, improved resistance against tomato bacterial speck disease caused by *Pseudomonas syringae* pv. *tomato* DC3000 [85]. Meanwhile, CRISPR/Cas-editing of the *SICLV3* promoter in tomatoes increased the fruit size and number of flower buds [86]. CRISPR/Cas9-based knockout of three pairs of *FAD2* homoeologs in the oilseed crop Camelina (*Camelina sativa*), increased monounsaturated fatty acids (MUFAs) by 80% with a stunted bushy phenotype, while transformants containing two pairs of *CsFAD2* homoeologs knocked out, and the other pair from the heterozygous wild-type, showed normal growth and seed MUFA levels increased by up to 60% [87]. Loss of seed shattering or seed dormancy was observed in canola by knocking out two *ALCATRAZ* genes [88]. Applications of other SSNs for crop improvement have also been reported, such as reduced browning in white button mushrooms (*Agaricus bisporus*) [89] and phytate in corn [55] using ZFN, and the creation of fragrant rice by disrupting the *OsBADH2* gene using TALEN, which resulted in the synthesis of 2-acetyl-1-pyrroline (2AP), a key fragrance compound [90]. 

Knocking out strategic genes through Type-1 modification could also increase crop resistance to various diseases, such as rice blast disease and citrus canker disease, by mutating the ethylene response factor (ERF) gene, *OsERF922*, in rice [91] and the canker susceptibility gene, *CsLOB1*, in grapefruit (*Citrus × paradisi*) [92], respectively. Furthermore, unlike the classical mutagenesis technique, Type-1 modification allows simultaneous alteration of multiple alleles from the same loci. This is particularly useful for polyploid plants in which the phenotype of the recessive mutation is usually hidden and only shows up in their progenies, requiring laborious crossing events to regain the desired traits. Examples include the use of TALENs in wheat to introduce multi-allelic DNA changes in the *Mildew Locus O (MLO)* region to establish a mildew-resistance trait [93], and the use of CRISPR/Cas9 to alter four different alleles from the gene encoding granule-bound starch synthase (GBSS) in tetraploid potato [94]. Overall, SSN-based genome editing for Type-1 modification has a relatively high success rate, with the recovery rate of plants with knockout gene(s) ranging from 2% to 75%, with a median of 25% [95,96].

Type-2 and Type-3 SSN-based modifications utilize the less frequent, albeit high-fidelity, HDR repair mechanism with strand invasion of an oligonucleotide or a ‘repair template’ containing the desired mutations (Figure 4). The repair template is homologous to the target site, and its length can vary from tens to hundreds of bases. Single or several base substitution is generally classified as Type-2 modification, whereas Type-3 modification involves larger DNA fragments or entire gene sequence introduction. Several herbicide-resistant GM crops have been generated by altering acetolactate synthase (ALS), a key enzyme targeted by many herbicides, with the variant degree of Type-2 modification, such as single point CRISPR/Cas9-mediated mutation in watermelon [97] and rice [98], double-point TALEN-mediated mutation in rice [99], and multiple points CRISPR/Cas9-mediated mutation in tomato [100]. Moreover, substituting thymine with adenine in position 317 of the *NON-RIPENING* (*NOR*) gene using CRISPR/Cas9-mediated mutation produced a longer shelf-life tomato than the wild-type [101]. In rice, TALEN-mediated depletion of the cytoplasmic male sterility-associated mitochondrial gene, *ORF312*, indicated that *ORF312* is a cytoplasmic male sterility (CMS)-causative gene [102].

Insertion in SSN-mediated Type-3 modification is precise, reducing the risk of genome disruption or positional effects. For example, Shi et al. [103] generated a highly productive and novel drought-tolerant corn variant by replacing the native promoter in the *ARGOS8* gene with a GOS2 promoter using an 800 bp repair template. Herbicide-resistant rice and cassava had also been generated through CRISPR/Cas9 SSN-mediated Type-3 modification using a 476 bp repair template to introduce multiple point mutations in the rice *ALS* gene [104] and a 4096 bp template to replace the cassava *ESPS* gene promoter with a strong constitutive 2 × 35S promoter [105]. So far, the largest insertion mediated by CRISPR/Cas9 editing was 5.2 kb encoding carotenoid biosynthesis in rice [106]. SSN-mediated gene insertion can facilitate gene stacking in crop improvement through ‘trait landing pads’ that allow the assembling of multiple transgenes at the same loci [107]. This allows the stack of genes to be passed down to the progenies in a single cassette during plant breeding, allowing easier introgression into desired elite lines. 

The inefficiency of gene replacement or gene insertion through innate HDR has remained a bottleneck to fully exploiting SSN-based genome editing for crop improvement. Few reports have utilized the more dominant NHEJ repair mechanism to achieve Type-2 modification by inducing two DSBs flanking the targeted sequence with a pair of SSNs. A repair template is supplied with ligation overhangs that are compatible with the two induced DSBs [96,108]. Another strategy is to fuse Cas9 endonuclease with *Agrobacterium* VirD2 relaxase, which brings the repair template in proximity to the DSB to increase the rate and efficiency of HDR [109]. In recent years, the cytosine and adenine base editor, mediated by CRISPR/Cas9, has emerged as an efficient target base editing tool to produce Type-2 modification for crop improvement [110,111]. In this ingenious innovation, the CRISPR/Cas9 was fused to the cytidine deaminase enzyme, which enabled direct single base substitution (C → T or G → A) without inducing DSB, eliminating the need for a repair template [112,113]. Adoption of this latest innovation has been swift and successful for the rapid generation of GM crops with herbicide-resistant traits in rice [114], corn [115], and canola [116], and impaired amylose biosynthesis in potato [117]. Nonetheless, several challenges that impair widespread base editing applications remain to be resolved, which have been reviewed by Bharat et al. [118].

Current research on SSN has focused on increasing its efficiency while minimizing off-target mutation. Simultaneously, there are continuous efforts to further innovate the tool by developing a DNA-free delivery system to eliminate the integration of transgenes that encode SSN-based components into the plant host genome. This could be achieved by delivering preassembled ribonucleoprotein complexes (RNPs), composed of purified recombinant enzyme Cas9 and in vitro-transcribed or synthesized guide RNA (gRNA), into plant protoplasts. Such a strategy was successfully demonstrated by Park et al. [119] in cabbage (*Brassica oleracea*) with a 2% mutation frequency. However, this approach could be extremely challenging in crop species with inefficient protoplast isolation and regeneration [96]. Another delivery method that has been reported was bombarding CRISPR/Cas9 RNPs into plant cells. A 4.4% mutation rate has been reported in bread wheat using this method [120]. Regardless of the strategy employed to achieve DNA-free genome editing, the goal is to overcome the hurdle of additional regulatory approval, especially in countries that rely on process-based regulation [121] and seek to minimize public concerns [106].

## 6. The Rapid Emergence of the CRISPR/Cas System for High Specificity Gene Editing

### 6.1. Variety of CRISPR Enzymes and Current Applications in Crops 

The discovery of CRISPR/Cas-based editing tools marked a major milestone for plant engineering [122]. First used in mammals, the CRISPR/Cas system involves pairing a gRNA and the CRISPR-associated (Cas) nuclease to recognize complementary nucleic acid sequences for cleavage. Successful targeting requires complementarity between the gRNA and the target site as well as a short sequence flanking region known as a protospacer-adjacent motif (PAM) [123,124]. Unlike ZFNs and TALENs, which require protein-DNA interaction and recoding of large DNA sequences (500–1500 bp) for each new target site, CRISPR-Cas9 is adaptable to many target sites. This is performed by simply changing and integrating the 20-bp protospacer of the guide RNA into the gRNA plasmid backbone while the Cas9 protein remains unaltered [124]. Due to its simplicity and efficiency, CRISPR systems have become the leading genome editing technology in various plant species, including model plants, food crops, industrial crops, and ornamental plants (Table 2). In addition, potential CRISPR applications for medicinal plants, such as *Salvia miltiorrhiza*, *Dendrobium officinale*, *Cannabis sativa*, and *Opium poppy*, have been proposed. However, they are still in the early stages of development [125]. 

Most mutation alterations induced by the CRISPR/Cas system to study gene functions are characterized by a small number of nucleotide insertions or deletions at the target site. For example, CRISPR/Cas9-mediated, 1-bp to 2-bp deletions in *Solanum lycopersicum SALT-RELATED MYB1-LIKE (SlSRM1-LIKE)* caused abnormal tomato leaf development with several morphological changes, including thinner leaves, wrinkled edges, raised veins, disordered edge veins, and left and right asymmetry [144]. A single base pair CG deletion in the *Hordeum vulgare* (barley) *MITOGEN-ACTIVATED PROTEIN KINASE6* (*HvMPK6*) using CRISPR/Cas9 led to severely reduced grain germination and abnormal seedlings with a shootless phenotype [145]. In addition, in barley, CRISPR/Cas9-mediated deletions ranging from 1 bp to 25 bp were detected during the double knockout of *Hordeum vulgare MICRORCHIDIA1* (*HvMORC1*) and *HvMORC6a*. Both genes play important roles in plant immunity and genome stability [146]. In corn, CRISPR/Cas9-mediated knockout of *Zea mays PHOSPHOLIPASE D3* (*ZmPLD3*) resulted in either 1-bp insertion or a combination of 1-bp insertion and 5-bp deletion in two separate mutant lines, which triggered haploid induction [147]. In rice, relatively larger deletions of 71 and 33 bp (rather than 1-bp deletion) were necessary to effectively knockout *Oryza sativa* MICRORNA168a (*OsMIR168a*), which caused significant transcription profile changes. This indicates *OsMIR168a*’s major transcriptional regulatory role, possibly through its potential target genes, such as *Oryza sativa ARGONAUTE1s* (*OsAGO1s*) and *OsAGO18* [148]. Potential phenotypic consequences of these transcription profile changes were investigated using KEGG enrichment analysis, suggesting *OsMIR168a*’s vital roles during plant growth and development as well as in plant–pathogen interaction [148]. 

Cas9 nuclease isolated from *Streptococcus pyogenes* (SpCas9) has been the most widely used in CRISPR/Cas systems [149]. However, SpCas9 has its disadvantages, such as its tendency to recognize DNA sequences with high similarity with the target site, causing off-target mutations [150,151,152]. Moreover, a limited number of DNA sequences can be altered with SpCas9 due to stringent NGG (N  =  A, T, C, or G) PAM requirements [153]. Additionally, the delivery of SpCas9 via a viral-based vector can be challenging due to its relatively large size, which exceeds the cargo size of the vector [154]. Several innovations have been made to overcome these challenges, such as substituting SpCas9 with a natural variant, *Staphylococcus aureus* Cas9 (SaCas9) which recognizes 5′-NNGRRT and has a shorter coding sequence [155]. Another improvement to the SpCas9 toolbox is the use of Cas9 nickase to increase binding specificity and reduce off-target DNA recognition [156]. Recently, PAM-less plant genome engineering has been established using an engineered SpRY Cas9 variant, which recognizes almost all PAM sites (NRN>NYN) [157]. Other SpCas9 variants have been developed, such as Cas9-NG [158], xCas9 [159,160], and iSpyMacCas9 [161], which have expanded the Cas–PAM compatibility, further improving the CRISPR/Cas toolbox for genome editing.

Cas12 nucleases are another family of Cas proteins applied in CRISPR/Cas-based plant genome editing. The Cas12 protein family, especially the Cas12a effector (formerly known as Cpf1), is considered a major improvement to the CRISPR/Cas system due to several characteristics: (1) smaller size [162], (2) lack of need for trans-activating crRNA (tracrRNA), (3) ability to cleave DNA via its RuvC domain, and (4) having intrinsic RNAse activity that can process its own guide RNA array, allowing multigene editing from a single RNA transcript. These traits further enhance CRISPR/Cas-based editing efficiency and specificity [162,163,164]. Moreover, further expansion of the PAM recognition range is currently explored with Cas12a orthologs, such as LbCas12a, AsCas12a, and FnCas12a and engineered variants, such as LbCas12a-E795L [165], AsCas12a Ultra [166], and LbCas12a-RVR [167]. So far, LbCas12a is the most well-known for its high efficiency in various crops, including Arabidopsis (*Arabidopsis thaliana*) [168,169,170], rice [171,172,173], corn [168,174], benthi (*Nicotiana benthamiana*) [169], tomato [169], lettuce (*Lactuca sativa*) [175], cotton [176,177], and citrus [178]. Although Cas9 is still the most routinely used nuclease in genome editing, Cas12a popularity is rapidly gaining momentum. Cas12a application has been shown to increase both NHEJ- and HDR-mediated editing efficiency due to the 5′ extension (4 to 25 nucleotides) of the multiple CRISPR RNAs (crRNAs) as a single-guide RNA (sgRNA) [179]. In rice, the use of both FnCas12a and LbCas12a, together with crRNA and repairing template DNA, is capable of mediating both NHEJ- and HDR-based genome editing [180]. In addition, in rice, the use of Cas12a, multiple crRNA, and donor repair templates in an all-in-one expression vector resulted in efficient HDR-mediated biallelic gene targeting within one generation [181]. This is a notable improvement compared to the predominant NHEJ-mediated monoallelic alteration, which usually resulted in a low mosaic recombination frequency in rice [182] and Arabidopsis [183]. As a newly identified nuclease for genome editing, continuous improvements have been made to the Cas12a system to be efficiently used for base editing [184,185] and transcriptional regulation alteration [184,186,187,188,189]. 

Another effector protein, C2c2 (later named Cas13a), targets 28 nucleotides downstream of crRNA, producing single-stranded RNA degradation and inhibiting the transcription of the pre-targeted genes. Cas13a has been used to modify rice with a maximal knockdown of 78% [190]. Cas13 has also been shown to significantly inhibit TMV in tobacco leaves, showing its potential use for disease resistance in crops [191]. Furthermore, modification of Cas13 (i.e., dCas13) allows safer genetic disease treatment in mammalian cells as RNA editing is better for recovery and post-transcriptional regulation than DNA editing [192,193,194]. In general, Cas13 provides more accurate silencing than RNAi. However, its application in crops is still being investigated [194]. 

### 6.2. CRISPR Reagents Delivery Systems

Delivery of CRISPR reagents into the plant host has been a major challenge due to the complex genome structure, polyploidy, and possible genomic rearrangements in plants [195]. Recent advancements in CRISPR delivery involve several methods, including Agrobacterium-mediated gene transfer, biolistic delivery, and the use of protoplasts as efficient systems for gene-editing and regeneration in plants.

One innovation in the Agrobacterium-mediated delivery system is to co-deliver developmental regulators (DR) with CRISPR components into the host plants [196]. The expression of DRs can be effectively used in plants that are hard to regenerate or have a long regeneration time [195]. Another interesting method involves using viral vectors as delivery vehicles for CRISPR/Cas components [197]. Adeno-associated viruses (AAVs) have been widely used for this purpose due to the high number of approved AAVs for human clinical trials and the fact that AAVs show fewer immunogenic effects than other viruses [198]. Other reasons for applying viral vectors include their ability to carry large DNA payloads and transduce a wide range of dividing and non-dividing cells [199]. 

Recently, an exciting discovery of a ‘hypercompact’ CRISPR-Casφ system (~70 kDa; half the size of Cas9 nuclease) by Pausch et al. [200] may allow gene editing by resolving cargo barriers of the positive-strand RNA virus (PSV). The PSV would carry a single guide RNA (sgRNA) with the Cas9 T-DNA construct. The sgRNA is expected to allow efficient germline invasion and collection of seeds with the heritable mutations, skipping the regeneration step in tissue culture [201]. 

Agrobacterium-mediated delivery and particle-based bombardment are two commonly used techniques to deliver CRISPR components into the plant host genome. The former involves the use of a binary vector, superbinary vector, and dual binary vector, and has recently progressed to the use of a ternary vector, further expediting the application of CRISPR-based plant genome editing [202]. On the other hand, particle-based bombardment involves using gold or tungsten particles coated with biomolecules that are physically inserted into the plant host genome via high-velocity bombardment using a gene gun or biolistic device [203]. Finally, protoplasts (plant cells without cell walls) offer a feasible DNA-free genome editing system. The protoplast system allows for the pre-evaluation of the gene-editing components before applying them to a full-scale transformation in the host plant.

Despite being a popular tool for genome editing, the CRISPR technology faces some technical challenges in delivering the CRISPR/Cas system into crop genomes. These include the low transformation efficiency and recalcitrance to the regeneration of several commercially important crops [204]. Hence, developing efficient and tissue-culture-independent delivery methods is indispensable.

## 7. SSN-based Genome Editing: Good or Bad?

SSN-based genome editing is critical for developing high-yielding, high-quality, and climate-resilient crops. However, despite significant contributions toward developing these crops, potential risks and ethical issues on the commercial release and consumption of genome-edited foods are still being debated.

The main benefit of SSN-based genome editing technologies is that these technologies could eliminate the transgene and produce crops with no difference from those generated from conventional breeding. These approaches might increase the acceptability of genome-edited crops and reduce ethical concerns about them. Furthermore, many efforts have been made to improve the efficiency of the SSN-based systems by uncovering new proteins and/or improving existing proteins in these systems.

Technical limitations of SSN-based genome editing technologies remain, however, creating significant concerns. These include the possibilities of low on-target editing efficiency and off-target and incomplete editing. For example, the presence of many identical target sites within a genome could affect the accuracy of the Cas9 endonuclease in targeting the correct location. Hence, improving the predictability of off-target editing is imperative to ensure strong public trust and the wider acceptability of genome-edited crops. Fortunately, significant efforts have been made to overcome these technical limitations, including improving delivery methods, methods for increasing the efficiency of DSB repair by HDR and precise gene regulation, and multiplexed and high-throughput genome editing approach.

Another concern is whether the edited crops will be affected indefinitely, or if the edited genes will be transferred to future generations, potentially affecting the crops in unexpected ways. The Cas9 and the sgRNA in the CRISPR/Cas9-based system are generally expressed from transgenes integrated into the plant genome [205]. Therefore, they must be removed because the presence of CRISPR/Cas9 might create challenges for the differentiation of previously generated mutations from newly generated ones. 

Genetic migration of the edited gene sequence from the genome-edited species to a wild-type species may have environmental consequences, thus limiting their adoption in agriculture [206]. However, whether SSN-based genome editing technologies are beneficial for crop improvement requires cultivating these edited crops in field conditions. Most previously reported genome-edited crops have not yet reached the field because of biosafety and regulatory issues. 

In 2021, the first CRISPR/Cas9-edited crop entered the market [58]. Known as the Sicilian Rouge High GABA, these CRISPR-edited tomatoes contain high levels of γ-aminobutyric acid (GABA), which is claimed to lower blood pressure and promote relaxation [62]. The commercialization of the CRISPR-edited tomato draws comparison with the previous release of the first RNAi-based ‘Flavr Savr’ tomato in 1994 in terms of their development, regulations, and public reception. Interestingly, the CRISPR-edited tomatoes were not regulated as GM food as they are ‘transgene-free’, therefore escaping the definition of a GM crop [207,208]. However, since the release of SSN-edited crops largely depends on the regulatory approval process, it is worth examining the current regulatory landscape governing genome-edited agricultural crop production.

## 8. SSN-based Genome Editing: A Modern Technology within a Conventional Regulatory Framework

Modern SSN-based genome editing allows more precise DNA alteration than conventional breeding techniques. Although off-target mutations in SSN-based genome editing have been reported, they involved few nucleotide changes within the same threshold of naturally-occurring mutations in plants [209]. On the other hand, mutation breeding techniques involving harmful rays or alkylating agents could introduce thousands of random DNA mutations across the entire plant genome [210]. The mutation breeding technique is considered a conventional method as it is supposed to mimic the natural process of mutations in plants and has been used to produce improved crop variants for almost a century [211]. To avoid unnecessary barriers imposed by stringent regulations, proponents argue that Type-1 and Type-2 SSN-based editing tools should be viewed as ‘precision breeding’ and properly be distinguished from the conventional DNA recombinant technology [212]. 

In the European Union (EU), a GMO is described in Directive 2001/18/EC as ‘an organism, except for human beings, in which the genetic material has been altered in a way that does not occur naturally by mating and/or natural recombination’. Therefore, even though mutation of DNA sequences that result from a genome editing technique and mutation breeding technique involves similar fundamental processes (induction of DSB and the activation of DNA repair machinery), only products from the latter technique and protoplast fusion are excluded from the definition of GMO. This was established by the recent ruling of the European Court of Justice (ECJ), which has confirmed that plants developed by genome editing approaches are covered by existing biosafety legislation. This includes the Directive 2001/18/EC, which implies that, because GM technology was involved in a plant’s development process, it should be subjected to a pre-market risk assessment according to the comprehensive general framework outlined in the Directive [80,213]. According to the ECJ, the risks of using new genome editing tools are possibly similar to those of GMOs resulting from classical mutagenesis techniques. They pointed out the lack of a long safety record due to the rapid introduction of novel products enabled by these new genome editing tools. In contrast, the US Department of Agriculture (USDA) has directed that plants that were developed using genome editing tools fall outside of its regulatory purview. This is because the plants do not contain any transgene from viruses or bacteria, which normally occur from the *Agrobacterium*-mediated transformation technique [214]. So far, five CRISPR-edited plants (soybean, camelina, corn, white button mushroom, and green bristlegrass) and several other TALEN-edited plants are already in development and have received USDA approval [214]. The ruling is significant because the unregulated genome-edited plants are no longer subjected to a lengthy and costly risk assessment evaluation.

Variations in the regulatory frameworks among many countries are expected as the regulatory processes are based on several factors, including the definition of living modified organism (LMO), the process of making the LMO-based product, and/or the risk associated with the intended use of the LMO-based product [121,215]. The two main protocols that are referred to for LMO definitions and concepts are (1) the Cartagena Protocol on Biosafety, and (2) Risk Analysis Principles for Foods Derived from Biotechnology (Codex Alimentarius Commission). According to the Cartagena Protocol, any plants produced as a result of genetic engineering are considered LMO, which is defined as ‘any living organism that processes a novel combination of genetic material obtained through the use of modern technology’. LMOs are also subjected to the risk analysis process, which should consider any hazardous, nutritional, or other safety concerns. In addition, the safety assessment should compare the food derived from modern biotechnology and its conventional counterpart to find any new or altered hazards, nutritional or other safety concerns [216]. Once the GMO is considered ‘substantially equivalent’ to its non-GMO counterpart, it is considered safe for food or feed consumption [217]. The LMO definition and the process involved in its development are the basis of many legal frameworks in the EU and other countries, such as Australia, New Zealand, Brazil, China, Japan, Saudi Arabia, and Thailand [218].

The EU probably has the strictest regulatory framework for conventional GM crop cultivation, which requires thorough environmental, human, and animal health safety assessments by the European Food Safety Authority (EFSA), as well as European Commission (EC) approval [207,219]. The insect-resistant corn, MON810 expressing Bt protein, Cry1Ab, was the only GM crop approved for cultivation in 1998 in the EU [220]. Despite positive recommendations from the EFSA, no other GM variants have been approved by the EC for cultivation, since most EU countries, including France and Germany, either partially or fully ban GMO cultivation [207]. Interestingly, despite its strong stance against GM crop cultivation, the EU is still one of the major importers of GM crops, including GM corn, cotton, soybean, canola, and sugar beet. The EU also depends on GM feed for their livestock industry, with an annual import of 30 million metric tons annually [221]. Pre-market assessments on transgene-stacked plants also vary between different countries. In the USA and Canada, a transgenic plant with stacked traits is not considered a new GMO event if the toxicological and allergenicity tests on the individual traits have been done before the stacking event [222]. However, in the EU and other countries, such as Argentina, Korea, and Japan, the same stacked gene product would still be considered a new and separate GMO event requiring an additional risk assessment, even if each trait had been pre-assessed before the stacking event [138,223,224,225].

As modern biotechnology tools become more complex, there have been renewed discussions on the current regulatory frameworks governing genetically engineered crop production. It has become crucial to acknowledge the different types of modification induced by SSN-based genome editing techniques within the LMO regulatory framework. Treating all types of SSN-mediated modifications within the same regulatory framework might hamper technological progress made in crop improvement. Among the three types of SSN-based modifications, Type-3 is the most similar to the conventional recombinant DNA technology which allows the replacement or addition of a whole gene, often foreign, in the plant host genome [226]. In contrast, Type-1 and Type-2 alter plant genomic sequences endogenously without the insertion of exogenous DNA, and, therefore, should not be regulated the same way as Type-3 modifications. Recently, the EFSA panels considered that the EU’s Guidance for risk assessment of food and feed from genetically modified plants and the Guidance on the environmental risk assessment of genetically modified plants were sufficient but should not be applicable to all plants produced via Type-1 and Type-2 editing. In addition, the panel members did not find any new hazards linked to Type-1 and Type-2 modifications when compared with Type-3 modification and conventional breeding [227]. 

A combination of various biotechnological approaches will give rise to GM crops with various traits, each one with a different level of risk [228]. Risk assessments on SSN-based products should be conducted on a case-by-case basis with great transparency by considering the processes involved in their development [80]. Similarly, a transparent regulatory framework is vital in gaining public trust. However, most of the existing LMO regulatory frameworks do not provide any means of ensuring an acceptable level of transparency [229]. Strauss and Sax [228] suggested that a registry of all GM products entering the market should be created. So far, the USDA’s Agricultural Marketing Service has published a list of GM crops and foods available throughout the world and for which the responsible agency must maintain a record (USDA Agricultural Marketing Service, 2021). Such registry should also include applications with differing regulatory statuses in various countries (e.g., SDN-1 in Argentina, Brazil, and the USA compared to the EU and New Zealand). Such transparency will help clarify the varying regulatory status of GM applications globally, improve international trade, and, hopefully, increase public understanding of GM products [229].

## 9. Benefits of Agricultural Biotechnology

### 9.1. Improved Crop Yield and Efficient Land Use

In the Green Revolution era, the widespread utilization of fertilizers and pesticides has significantly boosted agricultural production. Unfortunately, their usage as agricultural inputs has been reported to be a limiting factor towards the end of the Green Revolution era as the global yield has begun to plateau for some major cereal crops [230]. A prominent benefit of GM technology is its ability to increase crop yield within the same cultivation area with fewer inputs, mitigating the shrinking size of arable lands available for crop cultivation. According to the FAO, the arable land for food production per person will decrease from 0.24 ha in 2014 to only 0.18 ha in 2050. This does not include the additional usage of land to produce biofuel feedstock or the effects of urbanization or soil degradation [231]. Thus, there is a compelling need to produce higher agricultural yields by adopting GM technology to increase the food supply. 

The main impact of GM technology on improving crop yields has been through better weed control and reducing the damage caused by pests through the cultivation of herbicide-tolerant and insect-resistant crops. As a result, from 1996 to 2018, the application of GM technology has increased the global production of the main crops by producing an additional 498 million tons of corn, 278 million tons of soybean, 32.6 million tons of cotton lint, and 14.1 million tons of canola [232]. Without the cultivation of GM crops during this period, additional arable land of 12.3 million ha of soybeans, 8.1 million ha of corn, 3.1 million ha of cotton, and 0.7 million ha of canola would have been needed to cultivate the conventional crop equivalent [232]. This would have required the clearance of more areas from the tropical forests for cultivation and the use of more fertilizers, herbicides, and pesticides, as well as irrigation, to gain the same reported yield. The increase in crop yield is supported by a meta-analysis of 147 original studies from 1996 to 2014, which reported that GM technology had increased crop yields by 22%, with the yield gains larger for insect-resistant crops than for herbicide-tolerant crops [233]. 

Another interesting meta-analysis of data from 130 publications found that gene overexpression, or the ectopic expression of transporter genes or other gene types, in three major GM cereals (rice, wheat, and corn) had significantly increased the overall grain yield by 16.7% on average [234]. Studies on these GM crops have mainly focused on genes with probable essential roles in improving the nitrogen uptake efficiency of crops. One example is the overexpression of alanine aminotransferase (*AlaAT*) genes, which are responsible for the increase in nitrogen utilization efficiency (NUtE; the biomass or grain yield per unit of nitrogen uptake) in canola and rice [235,236]. Li et al. [234] further suggested that the increased yield in the GM crops might depend on the higher shoot biomass, nitrogen uptake efficiency (NUpE; plant roots capacity to acquire nitrogen from the soil), and partial factor productivity of nitrogen (PFPN; grain yield per unit of nitrogen applied in soil). In another meta-analysis study on peer-reviewed literature (from 1996 to 2016) on GM corn, the study found strong evidence of higher grain yield, ranging from 5.6 to 24.5%, higher than for the true non-GE or near-isogenic line [237]. The GM lines also contained lower concentrations of mycotoxins (~28.8%), fumonisin (~30.6%), and thricotecens (~36.5%) [237]. The evidence clearly shows the benefits of GM technology in improving crop yield and reducing the accumulation of harmful toxins in the grain. 

### 9.2. Economic Benefits to Farmers and Consumers

Through better management of weeds and pests and reduction in cost production, GM technology has significantly benefited farmers, with an additional gross income of USD 225.1 billion for the period 1996–2018 [232]. In 2018, most of the income benefits were earned by farmers in developing countries, where they received 53.7% of total income benefits, with an average of USD 4.41 received for each extra dollar invested in GM crop seeds [232]. This is consistent with previous studies showing that biotechnology applications in agriculture have brought economic benefits to numerous small-scale landholders in developing countries [238,239]. Moreover, GM technology not only benefits farmers and agribusinesses, but also consumers through lower costs of food supplies. It is conceivable that without the adoption of agricultural biotechnology that helped boost food supplies, commodity prices would have risen [240].

### 9.3. Reduced Environmental Impacts of Agriculture

The adoption of biotechnology in agriculture from 1996 to 2018 has lessened agriculture’s environmental impact by reducing pesticide spraying by 775 million kg, representing a global reduction of 8.3% [241]. A meta-analysis demonstrated an overall reduction in chemical pesticide use by 37% from 1996 to 2014 due to biotechnology adoption [233]. The shift from conventional tillage (CT) to reduced tillage or no-tillage (RT/NT) farming systems in the cultivation of GM crops has resulted in a further reduction in fuel use by 12,799 million liters which have led to a significant reduction in global greenhouse gas (GHG) emissions of 34,172 million kg of carbon dioxide. Consequently, soil quality was enhanced by the retention of about 302,364 million kg of carbon dioxide [241]. 

### 9.4. Increased Tolerance to Crop Diseases

The global crop yield loss due to emerging and re-emerging pests and diseases is relatively high and was estimated to be 21.5%, 30%, 22.5%, 17.2%, and 21.4% for wheat, rice, corn, potato, and soybean, respectively [242]. The food-deficit regions of the Indo-Gangetic Plain and sub-Saharan Africa are reported to have suffered the highest crop losses [242]. Since the development of virus-resistant tobacco expressing the TMV coat protein [37], various biotechnological strategies have been applied to confer disease resistance in crops. These strategies include intervention in pathogen recognition/perception, pathogen effector binding, altering the expression of genes in plant defense signaling, targeting recessive resistance traits/susceptibility genes, interspecies transfer of dominant plant resistance genes, and utilization of antimicrobial peptides and RNAi [243]. One of the most successful stories of biotechnological application in crops to mitigate virus infection is the papaya ringspot virus (PRSV)-resistant papaya, which can resist PRSV infection through the expression of a coat protein from PRSV [244]. The development of the transgenic cultivar successfully averted the devastating loss of the papaya industry caused by PRSV in Hawaii [245].

### 9.5. Nutrient Enhancement of Staple Crops

Staple crops, such as rice, contain low levels of beneficial phytonutrients (nutraceuticals) and micronutrients, often below the recommended daily allowance [246]. In 2020, it was estimated that nearly 10% of the world’s population (around 768 million people) were undernourished. More than half of all undernourished people live in Asia (418 million), while more than a third live in Africa (282 million) and a smaller proportion (60 million) in Latin America and the Caribbean [247]. Low- and middle-income countries rely more on starchy food staples, such as rice, banana (*Musa* spp.), cassava (*Manihot esculenta*), and corn. However, the majority are deficient in beneficial phytonutrients and micronutrients. The adoption of biotechnology is believed to offer an effective and sustainable strategy to produce GM biofortified crops with specific nutrient-enriched content. This is particularly important in countries where the technology is urgently needed to help alleviate nutrient-deficiency-related illnesses [248]. 

There has been considerable progress in developing biofortified staple crops, predominantly via synthetic metabolic engineering [249]. The best-known example, and the first biofortified staple crop utilizing this method, is the β-carotene-enriched ‘Golden Rice’ [250]. The bioavailability of the β-carotene in rice, a precursor of provitamin A, could reduce vitamin A deficiency, which affects an estimated 190 million preschool-age children worldwide, of whom 91.5 million reside in Southeast Asia [251]. The Golden Rice was produced by introducing the entire β-carotene biosynthetic pathway through the multigene transformation of rice endosperm on two T-DNAs [51]. The first T-DNA carried the daffodil (*Narcissus pseudonarcissus*) phytoene synthase gene, *NpPSY1*, and the bacterial (*Erwinia uredovora*) phytoene desaturase gene, *EuCRT1*, controlled by an endosperm-specific glutelin (Gt1) promoter and constitutive cauliflower mosaic virus (CaMV) 35S promoter, respectively. The second T-DNA carried the daffodil lycopene β-cyclase, *NpLYC-b* gene under the control of a rice glutelin promoter and a selectable marker. While the β-carotene enhancement in rice was successful, the carotenoid concentration was only increased by 1.6 μg/g dry weight (DW) [51]. This prompted the production of ‘Golden Rice 2′ (GR2), where the rice was engineered with corn *ZmPSY* and *E. uredovora EuCRTI* genes, both controlled by the native rice glutelin promoter [54]. The GR2 form contains a higher carotenoid accumulation of up to 23-fold (about 37 µg/g DW) than the original Golden Rice. Although GR2 is registered as safe in Australia, the USA, Canada, and New Zealand and possesses import approvals, the Philippines is the only country so far that has authorized the direct use of GR2 in food, feed, and processing. Since the production of GR2, increases in β-carotene levels through GM technology have been observed across an array of food crops, such as sorghum, corn, wheat, banana, and canola [252]. 

The successful generation of biofortified crops that involved the simultaneous expression of multigenes, with some generating multiple essential nutrients, was also reported. Enhancement of multiple vitamins, such as β-carotene, folate, and ascorbate, in the rice endosperm was achieved through the introduction of *ZmPSY1* and *EuCRTI* (carotenoid pathway), rice dehydroascorbate reductase, *OsDHAR* (ascorbate pathway), and *folE* (folate pathway) using an unlinked direct DNA transfer co-transformation strategy [253]. In a more recent study, multi-nutrient biofortified rice was developed by expressing *Arabidopsis thaliana NICOTIANAMINE SYNTHASE1* (*AtNAS1*), *Phaseolus vulgaris FERRITIN* (*PvFERRITIN*), bacterial *CRT1,* and *ZmPSY* in a single genetic locus that increased the levels of iron, zinc, and β-carotene content in the rice endosperm [254]. In an example that employed the multigene stacking strategy, the production of ‘second generation’ folate (Vitamin B_9_)-biofortified rice was achieved through simultaneous expression of four transgenes (*GTPCHI*, *ADCS*, *FPGS*, and *FBP*) [255]. Through this strategy, the folate content was increased significantly by 150-fold and has improved stability during post-harvest storage. Hence, this showed that the multigene stacking strategy is a highly efficient method for folate biofortification in rice, since the expression of a single transgene *GTPCHI* led only to about a 10-fold increase of folate concentration, whilst the co-expression of *GTPCHI* and *ADCS* resulted in a 100-fold folate enhancement [256].

### 9.6. Production of Plant-Based Pharmaceuticals

Global immunization coverage has declined from 86% in 2019 to 83% in 2020 due to the lack of access to immunization, aggravated by the straining of health systems due to the COVID-19 pandemic [257]. Children have been particularly affected, with the number of completely unvaccinated children increasing by 3.4 million in 2020 [257]. Vaccination coverage, specifically in underdeveloped countries, may be increased by developing a plant-based vaccine or ‘edible vaccine’. This innovation offers an exciting alternative by delivering a vaccine that can be easily administered and stored without refrigerated conditions [258]. An edible vaccine is produced by integrating specific genes encoding the desired antigenic protein into the plant host genome. Once the plant-derived vaccine is consumed, the release of antigens will stimulate a specific autoimmune response via mucosal immunity. Various candidates for plant-derived vaccines using economically important crops are currently under development, such as potato [259] and banana [260] expressing hepatitis B vaccine, tomato expressing triple vaccines against shigellosis, anthrax, and cholera [261], spinach (*Spinacea oleraceae*) expressing HIV-1 vaccine [262] and carrot (*Daucus carota*) expressing *Helicobacter pylori* vaccine [263]. An identical technique has been applied in chloroplast transformation, allowing a much higher accumulation of antigen protein because of the multi-copy nature of the chloroplast genome compared to the single-copy nature of the nuclear genome [264].

## 10. Concerns about the Effects of Agricultural Biotechnology on Human Health and the Environment

### 10.1. Effects on Human Health

Toxicity, allergenicity, and unintended genetic effects are three common concerns associated with GM food consumption. The causative factors may include: (i) integration of the transgene and the expressed protein per se, (ii) secondary and pleiotropic effects of the expressed gene, and (iii) insertional mutagenesis in the modified organism [46,265]. A well-known GM product linked to several allergenicity reports was ‘Starlink’ corn, which incorporated the *Bt* gene that produces an insecticidal Cry9c protein [266]. Due to the concern about the allergenic potential of Cry9c protein in humans, the EPA restricted the use of Starlink corn in animal feed consumption in 1998. However, its residues were later found in food products, where the Starlink was intermixed with corn in the food chain. The EPA received several reports of allergic reactions to corn products that may have contained Starlink, prompting the voluntary recall of several food products containing Starlink corn in 2000 [267,268]. However, subsequent epidemiological investigations by The Centers for Disease Control and Prevention (CDC) concluded that there was no evidence the allergenic reactions were associated with hypersensitivity to the Cry9c protein [269]. 

Secondary and pleiotropic effects through the expression of introduced transgenes, many of which encode enzymes, are harder to detect and recognize since there are no direct tests available. Such pleiotropic effects could be manifested as an alteration of normal flow rates of metabolites (decreases or increases) in the metabolic pathways of the modified organism [270]. However, the unintended effects of pleiotropy may be predicted based on available knowledge of the biochemical mechanism of the encoded protein. The likelihood of pleiotropy increases if the transgenes are involved in basic cellular functions or multiple biochemical pathways [270]. 

Another concern is the possible unintentional mutagenic effect outside the transgene insertion site. The disruption could possibly result in the inactivation of endogenous genes, or activation of otherwise silent genes that, in turn, may generate secondary toxic compounds, and the generation of fusion proteins [46]. While it is straightforward to test and confirm the direct effect of the transgene expression as shown with the Starlink Bt protein, it is harder to identify the causal connection between the transgene insertion and the toxic effect, particularly when the metabolic activation of the toxins are several steps away from the transgene insertion. 

Due to concerns about the possible transgene effects in transformed plants, the production of transgene-free plants is highly desirable but remains a challenge in plant biotechnology [271]. Current strategies to remove or prevent the integration of gene editor constructs in the CRISPR/Cas9 system involve the removal of transgenes via genetic segregation, transient editor expression from DNA vectors, and DNA-independent editor delivery [271]. A common genetic segregation system usually uses the target-site genotyping approach to isolate transgenic progenies from a population of transformed plants, followed by a counter-selection strategy using visible selection markers. In rice, a CRISPR/Cas9 containing a CYP81A6-hpRNAi expression cassette was used to produce bentazon (herbicide)-hypersensitive plants [272]. The T_1_ edited plants were then sprayed with bentazon, which killed the transgenic plants containing the transgenes, while the transgene-free rice seedlings grew normally [272]. Elimination of the transgene using marker gene selection is efficient. However, it raises other concerns (i.e., biosafety and biosecurity issues) regarding the use of antibiotic or herbicide-resistant transgenes in transformed crops. In the CRISPR/Cas system, transformed plants cannot survive without the transgene, requiring the screening of several generations to evaluate the presence of the transgene. 

Another widely used method in detecting transgene-free crops is the use of fluorescence-mediated monitoring to obtain transgene-free homozygous gene-edited crop plants [273]. In Arabidopsis, a CRISPR/Cas9 construct harboring the mCherry-expressing (red fluorescence) gene was used to visualize edited progenies in the T_1_ generation. This technique may save time in identifying transgene-free plants, which usually requires the laborious work of growing, extracting, and genotyping plant genomic DNA [274]. Another interesting approach is to use the transgene killer CRISPR (TKC) system, which allows automatic transgene elimination from the edited plants. In rice, a CRISPR/Cas9 construct harboring the self-eliminating genes, *barnase* and *CMS2*, which express toxic proteins during specific stages of plant development, was used to efficiently isolate transgene-free plants [275]. *Barnase* is driven by the host rice plant promoter, *REG2*, specifically during the embryogenic stage, while CMS2 interrupts mitochondrial functions in the callus and vegetative stages. This strategy allows the isolation of transgene-free plants during different growth stages of T_0_ plants [275]. 

### 10.2. Long Term Effects on Genetic Diversity

A wide genetic pool is vital for plant species to adapt to environmental changes. Classical and modern plant breeding techniques apply this knowledge by mixing different crop variants to create novel varieties with superior traits that would survive harsher conditions or produce higher yields. However, an estimated 75% of plant genetic diversity had diminished during the post-Green Revolution era as farmers around the world cultivated only a handful of high-yielding crop varieties in a mono-crop farming system to maximize profit [276]. The high cost of GM crop development imposed by the national and international regulatory processes has further narrowed down the biotechnological application to only high-value crops, such as soybean, corn, and cotton [228,277]. Over a long period, intensive cultivation, focusing on only a small number of crop species, will significantly affect the genetic pool and diversity, which is crucial for plant adaptation against climate change and virus and pest infestation [278,279]. 

### 10.3. Over-Reliance on Mega-Companies in the Agricultural Market

A major concern regarding GM crop cultivation is the over-reliance of farmers on large agro-biotech firms for patented seeds and agricultural chemicals. The reliance of farmers on these firms is compounded by the shrinking number of competitors in the market and the perceived oligopoly practice of a small group of mega-companies in the global agricultural market [280]. The merging of Bayer and Monsanto, for example, has resulted in Bayer owning 29% of seeds and 24% of pesticides in the global agriculture market in 2016 [281]. However, a meta-analysis of 15 studies demonstrated that two-thirds of profits of first-generation GM crops were shared downstream, while one-third accrued upstream by agribusiness companies [282], demonstrating that seed suppliers and large firms did not entirely dominate the profits. 

## 11. Conclusions

As agricultural production approaches a bottleneck due to limited arable lands, extreme weather, and increasing food demand [9], novel tools are needed to produce more resilient, efficient, and high-yielding crops to ensure global food security. Modern biotechnology tools with improved specificity and efficiency could eventually become the main driver of agricultural improvement, overcoming the limitations of conventional practices in improving crops. Rapid emergence and innovations in SSN-based editing tools especially show promising potential for crop improvement to boost agriculture productivity. Ultimately, transparency during the development, risk assessment, and regulatory process relating to modern biotechnology tools and the resulting products are crucial to further improve their applications in agriculture and increase public trust. Will the public accept SSN-based products more than GM products using DNA recombinant technology? Will modern biotechnology tools increase the pace of crop breeding substantially to improve food security? The answers to these questions will determine the fate of modern biotechnology tools in the agricultural sector and the lives of billions of people in the near future. 

## Figures and Tables

**Table 1:** Major milestones of biotechnological applications in agriculture.

Year	Milestone	References
~11,000 years ago	The oldest evidence of domestication of ‘founder crops’ (einkorn wheat, emmer wheat, barley, lentil, pea, chickpea, bitter vetch, flax).	[39]
1865	Gregor Mendel discovers the foundational principles of inheritance in a living organism by studying the common pea plant (*Pisum sativum*).	[40]
1897	Agrobacterium was first isolated from a crown gall tumor.	[41,42]
1898	The first documented study on the tobacco mosaic virus (TMV), laying the foundation of virology.	[43]
1907	The causative agent of the crown gall tumor was discovered and named *Bacterium tumefaciens*.	[44]
1940–1970 s	An in-depth study on the mechanism of crown gall tumorigenesis induced by *Agrobacterium tumefaciens*.	[35]
1983	The first transgenic plant was reported in tobacco (*Nicotiana tabacum*) harboring an antibiotic resistance gene.	[36]
1986	TMV-resistant transgenic tobacco was reported.	[37]
1987	Transgenic insect-resistant tobacco plant was reported.	[38]
1990	A ‘co-suppression’ phenomenon was observed in petunia (*Petunia hybrida*) that was genetically engineered to overexpress chalcone synthase (CHS). This started a new frontier in RNA interference (RNAi) research in living organisms.	[45]
1992	China became the first country to commercialize transgenic plants by introducing virus-resistant tobacco.	[46]
1993	The US Food and Drug Administration (FDA) approved the commercialization of the first transgenic food product, an RNAi-based ‘Flavr Savr’ tomato (cherry tomato; *Lycopersicon esculentum*).	[47]
1993	The European Union (EU) approved herbicide-resistant tobacco as the first genetically engineered crop to be commercialized in Europe.	[48]
1995	The US Environmental Protection Agency (EPA) approved the first pesticide-producing food crop (*Bacillus thuringiensis* [Bt] potato and Bt corn) and non-food crop (Bt cotton).	[49]
1996	Glyphosate-resistant soybean (*Glycine max*) became the first herbicide-resistant crop to be marketed for the consumer market in the US.	[50]
2000	Biofortified rice, known as ‘Golden Rice’, successfully demonstrated that engineering an entire biosynthetic pathway in an organism was possible.	[51]
2000	The first plant genome sequence was reported in Arabidopsis.	[52]
2005	The rice genome became the first crop plant to be sequenced.	[53]
2005	Golden Rice 2 with an increase in total carotenoids of up to 23-fold was reported.	[54]
2009	The first report of zinc-finger nuclease (ZFN) application in plants (corn).	[55]
2012	The first report of transcription activator-like effector (TALENS) application in plants (rice).	[56]
2013	The first report of clustered regularly interspersed short palindromic repeats (CRISPR) application in plants (rice and common wheat).	[57]
2021	First commercialization of a CRISPR-edited crop (tomato).	[58]

**Table 2:** CRISPR/Cas applications in various plants.

Crop	Target Site	Result	Reference
**Model plants**			
Arabidopsis *(Arabidopsis thaliana*)	Transgene mutant *GFP*	Insertion and deletion mutations at the targeted 20 bp sequences; restoration of GFP functionality	[126]
Rice *(Oryza sativa*)	Promoter region of the bacterial blight susceptibility genes, *OsSWEET14* and *OsSWEET11*	Deletion and substitution mutations	[126]
Tobacco (*Nicotiana tabacum*)	Transgene mutant *GFP*	Insertion and deletion mutations at the targeted 20 bp sequences; restoration of GFP functionality	[126]
Benthi (*Nicotiana benthamiana*)	Nuclear-localization (PDS locus) of GFP-Cas9 expression	Deletion and substitution mutations	[127]
**Food crops**			
Corn (maize; *Zea mays*)	Upstream of the *LIGULELESS1* (*LIG1*), male fertility genes (*Ms26* and *Ms45*), and acetolactate synthase genes (*ALS1* and *ALS2*)	*ALS2* editing yielded chlorsulfuron-resistant plants	[128]
Tomato (*Solanum lycopersicum*)	*ANTHOCYANIN MUTANT1* (*ANT1*)	*ANT1*-overexpression, which encodes a Myb transcription factor, results in intensely purple plant tissue due to anthocyanin accumulation	[129]
Sorghum *(Sorghum bicolor*)	An out-of-frame red fluorescence protein gene (DsRED2)	Restoration of DsRED2 fluorescence	[126]
Soybean (*Glycine max*)	Transgene *GFP*; single-copy soybean gene, Glyma07g14530; homoeologous gene-pair, Glyma01g38150 and Glyma11g07220; homoeologous gene pair Glyma04g36150 and Glyma06g18790; soybean miRNAs, miR1514 and miR1509	Loss of GFP fluorescence; variety of mutations, including deletions, SNPs, insertions, and replacements (two or more bases inserted after a deletion event)	[130]
Potato (*Solanum tuberosum*)	*StIAA2* encoding an Aux/IAA protein involved in petiole hyponasty and shoot morphogenesis	Deletion, insertion, and substitution mutations	[131]
Kiwifruit (*Actinidia chinensis*)	*CENTRORADIALIS* (*CEN*)-like genes, *AcCEN4* and *AcCEN*	Transformed a climbing woody perennial into a compact plant with rapid terminal flower and fruit development	[132]
Banana (*Musa balbisiana*)	Integrated endogenous banana streak virus (eBSV) sequences	eBSV knockout	[133]
Wheat (*Tricium aestivum*)	*TaMLO*	Insertion and deletion mutations frequencies of 26.5–38.0%	[57]
**Industrial crops**			
Poplar (*Populus tomentosa*)	*Populus tomentosa PHYTOENE DESATURASE GENE8* (*PtoPDS*) required for chlorophyll biosynthesis	Mutants with albino phenotype	[134]
Canola or rapeseed (*Brassica napus*)	*ALCATRAZ* (*ALC*). *ALC* is involved in valve margin development, therefore contributes to seed shattering from mature fruits	Increased shatter resistance (avoid seed loss during mechanical harvest)	[88]
Cotton (*Gossypium hirsutum*)	*GhMYB25-like A* and *GhMYB25-like D*	Deletion mutations of −1bp/−3bp/−7bp nucleotides and +1 bp insertion mutation; an indication of efficient genomic editing in the allotetraploid cotton genome	[135]
Rubber tree (*Hevea brasiliensis*)	*FLOWERING LOCUS T* (*FT*) and *TERMINAL FLOWER1* (*TFL1*)	Mutation frequencies ranging from 3.74% to 20.11% at five target sites; Insertion and deletion patterns	[136]
Oil palm (*Elaeis guineensis*)	*Elaeis guineensis PHYTOENE DESATURASE* (*EgPDS*)	Insertions, deletions, and nucleotide substitutions, with a mutation efficiency of 62.5–83.33%; chimeric albino phenotypes	[137]
Moso bamboo (*Phyllostachys edulis*)	*PePDS1* and *PePDS2*	Insertion and deletion mutations; mutants with albino shoot phenotype	[138]
**Ornamental plants**			
Indian chrysanthemum (*Chrysanthemum nankingense*)	Integrated *Chiridius poppei* (*CpYGFP*) expressing yellowish-green fluorescent protein	Mostly small deletions (1 bp); a large deletion (−1020 bp) was also observed	[139]
Japanese morning glory (*Ipomoea nil*)	*EPHEMERAL1* (*EPH1*) crucial in petal senescence	1-bp and/or 2-bp deletions occurred at the target sites	[140]
Coral lily (*Lilium pumilum*) and Easter lily (*Lilium longiflorum*)	*PDS*	Insertion, deletion and substitution; Mutants with completely albino, pale yellow and albino–green chimeric phenotypes	[141]
Petunia (*Petunia hybrida*)	*DEEP PURPLE* (*DPL*)	Insertion and deletion mutations; absence of the vein-associated anthocyanin pattern above the abaxial surface of the flower bud, but not corolla tube venation	[142]
Orchid (*Phalaenopsis equestris*)	*MADS44*, *MADS36* and *MADS8*	Insertion and deletion	[143]

## Data Availability

Not applicable.

## References

[1] The Food and Agriculture Organization (FAO) (2018). The Future of Food and Agriculture: Alternative Pathways to 2050.

[2] The Food and Agriculture Organization (FAO) Small Family Farmers Produce a Third of the World’s Food. https://www.fao.org/newsroom/detail/Small-family-farmers-produce-a-third-of-the-world-s-food/en.

[3] The Food and Agriculture Organization (FAO) Staple Foods: What Do People Eat?. https://www.fao.org/3/u8480e/u8480e07.htm#:~:text=The%20world%20has%20over%2050,animal%20products%20(7%20percent).

[4] The International Service for the Acquisition of Agri-biotech Applications (ISAAA) Pocket K No. 13: Conventional Plant Breeding. https://www.isaaa.org/resources/publications/pocketk/13/default.asp.

[5] Ania W., Mark W. History of Agricultural Biotechnology: How Crop Development has Evolved. https://www.nature.com/scitable/knowledge/library/history-of-agricultural-biotechnology-how-crop-development-25885295/.

[6] Orton T.J., Orton T.J. (2020). Chapter 12—protection of proprietary plant germplasm. Horticultural Plant Breeding.

[7] Pingali P.L. (2012). Green revolution: Impacts, limits, and the path ahead. Proc. Natl. Acad. Sci. USA.

[8] van Dijk M., Morley T., Rau M.L., Saghai Y. (2021). A meta-analysis of projected global food demand and population at risk of hunger for the period 2010–2050. Nat. Food.

[9] United Nations (UN) (2019). World Population Prospects 2019: Highlights.

[10] Karunarathna N.L., Patiranage D.S.R., Harloff H.J., Sashidhar N., Jung C. (2021). Genomic background selection to reduce the mutation load after random mutagenesis. Sci. Rep..

[11] Bailey-Serres J., Parker J.E., Ainsworth E.A., Oldroyd G.E.D., Schroeder J.I. (2019). Genetic strategies for improving crop yields. Nature.

[12] Viana V.E., Pegoraro C., Busanello C., Costa de Oliveira A. (2019). Mutagenesis in rice: The basis for breeding a new super plant. Front. Plant Sci..

[13] Muller H.J. (1927). Artificial transmutation of the gene. Science.

[14] Stadler L.J. (1928). Mutations in barley induced by X-rays and radium. Science.

[15] Stadler L.J. (1930). Recovery following genetic deficiency in maize. Proc. Natl. Acad. Sci. USA.

[16] Pacher M., Puchta H. (2017). From classical mutagenesis to nuclease-based breeding—directing natural DNA repair for a natural end-product. Plant J..

[17] Mei M., Deng H., Lu Y., Zhuang C., Liu Z., Qiu Q., Qiu Y., Yang T.C. (1994). Mutagenic effects of heavy ion radiation in plants. Adv. Space Res..

[18] Dhaliwal A.K., Mohan A., Sidhu G., Maqbool R., Gill K.S. (2015). An ethylmethane sulfonate mutant resource in pre-green revolution hexaploid wheat. PLoS ONE.

[19] Kumawat S., Rana N., Bansal R., Vishwakarma G., Mehetre S.T., Das B.K., Kumar M., Kumar Yadav S., Sonah H., Sharma T.R. (2019). Expanding avenue of fast neutron mediated mutagenesis for crop improvement. Plants.

[20] Kambhampati S., Aznar-Moreno J.A., Hostetler C., Caso T., Bailey S.R., Hubbard A.H., Durrett T.P., Allen D.K. (2019). On the inverse correlation of protein and oil: Examining the effects of altered central carbon metabolism on seed composition using soybean fast neutron mutants. Metabolites.

[21] Islam N., Krishnan H.B., Natarajan S. (2020). Proteomic profiling of fast neutron-induced soybean mutant unveiled pathways associated with increased seed protein content. J. Proteome Res..

[22] Holme I.B., Gregersen P.L., Brinch-Pedersen H. (2019). Induced genetic variation in crop plants by random or targeted mutagenesis: Convergence and differences. Front. Plant Sci..

[23] Kaiser N., Douches D., Dhingra A., Glenn K.C., Herzig P.R., Stowe E.C., Swarup S. (2020). The role of conventional plant breeding in ensuring safe levels of naturally occurring toxins in food crops. Trends Food Sci. Technol..

[24] López-Caamal A., Tovar-Sánchez E. (2014). Genetic, morphological, and chemical patterns of plant hybridization. Rev. Chil. Hist. Nat..

[25] Charlesworth D. (2006). Evolution of plant breeding systems. Curr. Biol..

[26] Vallejo-Marin M., Walker C., Friston-Reilly P., Solis-Montero L., Igic B. (2014). Recurrent modification of floral morphology in heterantherous solanum reveals a parallel shift in reproductive strategy. Philos. Trans. R. Soc. Lond. B. Biol. Sci..

[27] Erickson L.R., Atnaseo C., Moo-Young M. (2011). 4.10—transgenic crops with producer-oriented traits: Development, application, and impact. Comprehensive Biotechnology.

[28] Ghețe A.B., Haș V., Vidican R., Copândean A., Ranta O., Moldovan C.M., Crișan I., Duda M.M. (2020). Influence of detasseling methods on seed yield of some parent inbred lines of turda maize hybrids. Agronomy.

[29] Yu D., Gu X., Zhang S., Dong S., Miao H., Gebretsadik K., Bo K. (2021). Molecular basis of heterosis and related breeding strategies reveal its importance in vegetable breeding. Hortic. Res..

[30] Leducq J.B., Gosset C.C., Gries R., Calin K., Schmitt E., Castric V., Vekemans X. (2014). Self-incompatibility in Brassicaceae: Identification and characterization of *SRK*-like sequences linked to the *S*-locus in the tribe Biscutelleae. G3 Genes Genomes Genet..

[31] Xu F., Yang X., Zhao N., Hu Z., Mackenzie S.A., Zhang M., Yang J. (2022). Exploiting sterility and fertility variation in cytoplasmic male sterile vegetable crops. Hortic. Res..

[32] Munoz-Sanz J.V., Zuriaga E., Cruz-Garcia F., McClure B., Romero C. (2020). Self-(in)compatibility systems: Target traits for crop-production, plant breeding, and biotechnology. Front. Plant Sci..

[33] Wu F., Butz P.W. (2004). The gene revolution genetically modified crops. The Future of Genetically Modified Crops.

[34] Cohen S.N., Chang A.C., Boyer H.W., Helling R.B. (1973). Construction of biologically functional bacterial plasmids in vitro. Proc. Natl. Acad. Sci. USA.

[35] Kado C.I. (2014). Historical account on gaining insights on the mechanism of crown gall tumorigenesis induced by *Agrobacterium Tumefaciens Front*. Microbiol..

[36] Bevan M.W., Flavell R.B., Chilton M.D. (1983). A chimaeric antibiotic resistance gene as a selectable marker for plant cell transformation. Biotechnology.

[37] Abel P.P., Nelson R.S., De B., Hoffmann N., Rogers S.G., Fraley R.T., Beachy R.N. (1986). Delay of disease development in transgenic plants that express the tobacco mosaic virus coat protein gene. Science.

[38] Vaeck M., Reynaerts A., Höfte H., Jansens S., De Beuckeleer M., Dean C., Zabeau M., Montagu M.V., Leemans J. (1987). Transgenic plants protected from insect attack. Nature.

[39] Rottenberg A. (2017). Has agriculture dispersed worldwide from a single origin?. Genet. Resour. Crop Evol..

[40] Mendel G. (1866). Verhandlungen des naturforschenden vereines zu brünn. Verh. Naturf. Ver. Brünn.

[41] Cavara F. (1897). Eziologia di alcune malattie di piante coltivate. Le Stazioni Sper. Agrar. Ital..

[42] Cavara F. (1897). Tuberculosi della vite. Intorno alla eziologia di alcune malattie di piante cultivate. Le Stazioni Sper. Agrar. Ital..

[43] Sankaran N. (2018). On the historical significance of Beijerinck and his *contagium vivum fluidum* for modern virology. Hist. Philos. Life Sci..

[44] Smith E.F., Townsend C.O. (1907). A plant-tumor of bacterial origin. Science.

[45] Napoli C., Lemieux C., Jorgensen R. (1990). Introduction of a chimeric chalcone synthase gene into petunia results in reversible co-suppression of homologous genes in trans. Plant Cell.

[46] Bawa A.S., Anilakumar K.R. (2013). Genetically modified foods: Safety, risks and public concerns-a review. J. Food Sci. Technol..

[47] Kramer M.G., Redenbaugh K. (1994). Commercialization of a tomato with an antisense polygalacturonase gene: The FLAVR SAVR™ tomato story. Euphytica.

[48] Abbas M.S.T. (2018). Genetically engineered (modified) crops (*Bacillus thuringiensis* crops) and the world controversy on their safety. Egypt. J. Biol. Pest Control.

[49] Sanahuja G., Banakar R., Twyman R.M., Capell T., Christou P. (2011). *Bacillus thuringiensis*: A century of research, development and commercial applications. Plant Biotechnol. J..

[50] Dill G.M. (2005). Glyphosate-resistant crops: History, status and future. Pest Manag Sci..

[51] Ye X., Al-Babili S., Klöti A., Zhang J., Lucca P., Beyer P., Potrykus I. (2000). Engineering the provitamin a (beta-carotene) biosynthetic pathway into (carotenoid-free) rice endosperm. Science.

[52] Adjusted Gross Income (AGI) (2000). Analysis of the genome sequence of the flowering plant *Arabidopsis thaliana*. Nature.

[53] The International Rice Genome Sequencing Project *(*IRGSP*)* (2005). The map-based sequence of the rice genome. Nature.

[54] Paine J.A., Shipton C.A., Chaggar S., Howells R.M., Kennedy M.J., Vernon G., Wright S.Y., Hinchliffe E., Adams J.L., Silverstone A.L. (2005). Improving the nutritional value of golden rice through increased pro-vitamin a content. Nat. Biotechnol..

[55] Shukla V.K., Doyon Y., Miller J.C., DeKelver R.C., Moehle E.A., Worden S.E., Mitchell J.C., Arnold N.L., Gopalan S., Meng X. (2009). Precise genome modification in the crop species *Zea mays* using zinc-finger nucleases. Nature.

[56] Li T., Liu B., Spalding M.H., Weeks D.P., Yang B. (2012). High-efficiency TALEN-based gene editing produces disease-resistant rice. Nat. Biotechnol..

[57] Shan Q., Wang Y., Li J., Zhang Y., Chen K., Liang Z., Zhang K., Liu J., Xi J.J., Qiu J.L. (2013). Targeted genome modification of crop plants using a CRISPR-Cas system. Nat. Biotechnol..

[58] Waltz E. (2022). GABA-enriched tomato is first CRISPR-edited food to enter market. Nat. Biotechnol..

[59] Lindbo J.A., Silva-Rosales L., Proebsting W.M., Dougherty W.G. (1993). Induction of a highly specific antiviral state in transgenic plants: Implications for regulation of gene expression and virus resistance. Plant Cell.

[60] Vega Rodríguez A., Rodríguez-Oramas C., Sanjuán Velázquez E., Hardisson de la Torre A., Rubio Armendáriz C., Carrascosa Iruzubieta C. (2022). Myths and realities about genetically modified food: A risk-benefit analysis. Appl. Sci..

[61] James C., Krattiger A.F. (1996). Global Review of the Field Testing and Commercialization of Transgenic Plants, 1986 to 1995: The First Decade of Crop Biotechnology.

[62] The International Service for the Acquisition of Agri-biotech Applications (ISAAA) Japan Starts Sale of Genome-Edited High-GABA Tomato. https://www.isaaa.org/kc/cropbiotechupdate/article/default.asp?ID=19024.

[63] Que Q., Chilton M.D., de Fontes C.M., He C., Nuccio M., Zhu T., Wu Y., Chen J.S., Shi L. (2010). Trait stacking in transgenic crops: Challenges and opportunities. GM Crops.

[64] Chen W., Ow D.W. (2017). Precise, flexible and affordable gene stacking for crop improvement. Bioengineered.

[65] Das G., Patra J.K., Baek K.H. (2017). Insight into mas: A molecular tool for development of stress resistant and quality of rice through gene stacking. Front. Plant Sci..

[66] Collier R., Thomson J.G., Thilmony R. (2018). A versatile and robust Agrobacterium-based gene stacking system generates high-quality transgenic Arabidopsis plants. Plant J..

[67] Affoh R., Zheng H., Dangui K., Dissani B.M. (2022). The impact of climate variability and change on food security in sub-saharan africa: Perspective from panel data analysis. Sustainability.

[68] Dasgupta S., Robinson E.J.Z. (2022). Attributing changes in food insecurity to a changing climate. Sci. Rep..

[69] Agrimonti C., Lauro M., Visioli G. (2021). Smart agriculture for food quality: Facing climate change in the 21st century. Crit. Rev. Food Sci. Nutr..

[70] Kassaye A.Y., Shao G., Wang X., Shifaw E., Wu S. (2021). Impact of climate change on the staple food crops yield in ethiopia: Implications for food security. Theor. Appl. Climatol..

[71] Gao C. (2021). Genome engineering for crop improvement and future agriculture. Cell.

[72] Jaganathan D., Ramasamy K., Sellamuthu G., Jayabalan S., Venkataraman G. (2018). CRISPR for crop improvement: An update review. Front. Plant Sci..

[73] Dong O.X., Ronald P.C. (2021). Targeted DNA insertion in plants. Proc. Natl. Acad. Sci. USA.

[74] Rasheed A., Gill R.A., Hassan M.U., Mahmood A., Qari S., Zaman Q.U., Ilyas M., Aamer M., Batool M., Li H. (2021). A critical review: Recent advancements in the use of CRISPR/Cas9 technology to enhance crops and alleviate global food crises. Curr. Issues Mol. Biol..

[75] Iqbal Z., Iqbal M.S., Ahmad A., Memon A.G., Ansari M.I. (2020). New prospects on the horizon: Genome editing to engineer plants for desirable traits. Curr. Plant Biol..

[76] Karlson C.K.S., Mohd-Noor S.N., Nolte N., Tan B.C. (2021). CRISPR/dcas9-based systems: Mechanisms and applications in plant sciences. Plants.

[77] Ranjha L., Howard S.M., Cejka P. (2018). Main steps in DNA double-strand break repair: An introduction to homologous recombination and related processes. Chromosoma.

[78] van Gent D.C., van der Burg M. (2007). Non-homologous end-joining, a sticky affair. Oncogene.

[79] Lusser M., Parisi C., Plan D., Rodríguez-Cerezo E. (2011). New Plant Breeding Techniques: State-of-the-Art and Prospects for Commercial Development.

[80] Eckerstorfer M.F., Dolezel M., Heissenberger A., Miklau M., Reichenbecher W., Steinbrecher R.A., Wassmann F. (2019). An EU perspective on biosafety considerations for plants developed by genome editing and other new genetic modification techniques (nGMs). Front. Bioeng. Biotechnol..

[81] Kieu N.P., Lenman M., Wang E.S., Petersen B.L., Andreasson E. (2021). Mutations introduced in susceptibility genes through CRISPR/Cas9 genome editing confer increased late blight resistance in potatoes. Sci. Rep..

[82] Oliva R., Ji C., Atienza-Grande G., Huguet-Tapia J.C., Perez-Quintero A., Li T., Eom J.S., Li C., Nguyen H., Liu B. (2019). Broad-spectrum resistance to bacterial blight in rice using genome editing. Nat. Biotechnol..

[83] Xie K., Yang Y. (2013). RNA-guided genome editing in plants using a CRISPR-Cas system. Mol. Plant.

[84] Li M., Li X., Zhou Z., Wu P., Fang M., Pan X., Lin Q., Luo W., Wu G., Li H. (2016). Reassessment of the four yield-related genes Gn1a, DEP1, GS3, and IPA1 in rice using a CRISPR/Cas9 system. Front. Plant Sci..

[85] Ortigosa A., Gimenez-Ibanez S., Leonhardt N., Solano R. (2019). Design of a bacterial speck resistant tomato by CRISPR/Cas9-mediated editing of *SlJAZ2*. Plant Biotechnol. J..

[86] Rodriguez-Leal D., Lemmon Z.H., Man J., Bartlett M.E., Lippman Z.B. (2017). Engineering quantitative trait variation for crop improvement by genome editing. Cell.

[87] Lee K.R., Jeon I., Yu H., Kim S.G., Kim H.S., Ahn S.J., Lee J., Lee S.K., Kim H.U. (2021). Increasing monounsaturated fatty acid contents in hexaploid camelina sativa seed oil by *FAD2* gene knockout using CRISPR-Cas9. Front. Plant Sci..

[88] Braatz J., Harloff H.J., Mascher M., Stein N., Himmelbach A., Jung C. (2017). CRISPR-Cas9 targeted mutagenesis leads to simultaneous modification of different homoeologous gene copies in polyploid oilseed rape (*Brassica napus*). Plant Physiol..

[89] Waltz E. (2016). Gene-edited CRISPR mushroom escapes us regulation. Nature.

[90] Shan Q., Zhang Y., Chen K., Zhang K., Gao C. (2015). Creation of fragrant rice by targeted knockout of the *OsBADH2* gene using TALEN technology. Plant Biotechnol. J..

[91] Wang F., Wang C., Liu P., Lei C., Hao W., Gao Y., Liu Y.G., Zhao K. (2016). Enhanced rice blast resistance by CRISPR/Cas9-targeted mutagenesis of the ERF transcription factor gene *OsERF922*. PLoS ONE.

[92] Jia H., Zhang Y., Orbovic V., Xu J., White F.F., Jones J.B., Wang N. (2017). Genome editing of the disease susceptibility gene *CsLOB1* in citrus confers resistance to citrus canker. Plant Biotechnol. J..

[93] Wang Y., Cheng X., Shan Q., Zhang Y., Liu J., Gao C., Qiu J.L. (2014). Simultaneous editing of three homoeoalleles in hexaploid bread wheat confers heritable resistance to powdery mildew. Nat. Biotechnol..

[94] Andersson M., Turesson H., Nicolia A., Falt A.S., Samuelsson M., Hofvander P. (2017). Efficient targeted multiallelic mutagenesis in tetraploid potato (*Solanum tuberosum*) by transient CRISPR-Cas9 expression in protoplasts. Plant Cell Rep..

[95] Zhu C., Bortesi L., Baysal C., Twyman R.M., Fischer R., Capell T., Schillberg S., Christou P. (2017). Characteristics of genome editing mutations in cereal crops. Trends Plant Sci..

[96] Ran Y., Patron N., Kay P., Wong D., Buchanan M., Cao Y.Y., Sawbridge T., Davies J.P., Mason J., Webb S.R. (2018). Zinc finger nuclease-mediated precision genome editing of an endogenous gene in hexaploid bread wheat (*Triticum aestivum*) using a DNA repair template. Plant Biotechnol. J..

[97] Tian S., Jiang L., Cui X., Zhang J., Guo S., Li M., Zhang H., Ren Y., Gong G., Zong M. (2018). Engineering herbicide-resistant watermelon variety through CRISPR/Cas9-mediated base-editing. Plant Cell Rep..

[98] Wang F., Xu Y., Li W., Chen Z., Wang J., Fan F.-j., Tao Y., Jiang Y., Zhu Q.-H., Yang J. (2021). Creating a novel herbicide-tolerance *OsALS* allele using CRISPR/Cas9-mediated gene editing. Crop J..

[99] Li T., Liu B., Chen C.Y., Yang B. (2016). TALEN-mediated homologous recombination produces site-directed DNA base change and herbicide-resistant rice. J. Genet. Genom..

[100] Danilo B., Perrot L., Mara K., Botton E., Nogue F., Mazier M. (2019). Efficient and transgene-free gene targeting using Agrobacterium-mediated delivery of the CRISPR/Cas9 system in tomato. Plant Cell Rep..

[101] Yu Q.H., Wang B., Li N., Tang Y., Yang S., Yang T., Xu J., Guo C., Yan P., Wang Q. (2017). CRISPR/Cas9-induced targeted mutagenesis and gene replacement to generate long-shelf life tomato lines. Sci. Rep..

[102] Takatsuka A., Kazama T., Arimura S.I., Toriyama K. (2022). TALEN-mediated depletion of the mitochondrial gene orf312 proves that it is a Tadukan-type cytoplasmic male sterility-causative gene in rice. Plant J..

[103] Shi J., Gao H., Wang H., Lafitte H.R., Archibald R.L., Yang M., Hakimi S.M., Mo H., Habben J.E. (2017). ARGOS8 variants generated by CRISPR-Cas9 improve maize grain yield under field drought stress conditions. Plant Biotechnol. J..

[104] Sun Y., Zhang X., Wu C., He Y., Ma Y., Hou H., Guo X., Du W., Zhao Y., Xia L. (2016). Engineering herbicide-resistant rice plants through CRISPR/Cas9-mediated homologous recombination of acetolactate synthase. Mol. Plant.

[105] Hummel A.W., Chauhan R.D., Cermak T., Mutka A.M., Vijayaraghavan A., Boyher A., Starker C.G., Bart R., Voytas D.F., Taylor N.J. (2018). Allele exchange at the EPSPS locus confers glyphosate tolerance in cassava. Plant Biotechnol. J..

[106] Dong O.X., Yu S., Jain R., Zhang N., Duong P.Q., Butler C., Li Y., Lipzen A., Martin J.A., Barry K.W. (2020). Marker-free carotenoid-enriched rice generated through targeted gene insertion using CRISPR-Cas9. Nat. Commun..

[107] Ainley W.M., Sastry-Dent L., Welter M.E., Murray M.G., Zeitler B., Amora R., Corbin D.R., Miles R.R., Arnold N.L., Strange T.L. (2013). Trait stacking via targeted genome editing. Plant Biotechnol. J..

[108] Bonawitz N.D., Ainley W.M., Itaya A., Chennareddy S.R., Cicak T., Effinger K., Jiang K., Mall T.K., Marri P.R., Samuel J.P. (2019). Zinc finger nuclease-mediated targeting of multiple transgenes to an endogenous soybean genomic locus via non-homologous end joining. Plant Biotechnol. J..

[109] Ali Z., Shami A., Sedeek K., Kamel R., Alhabsi A., Tehseen M., Hassan N., Butt H., Kababji A., Hamdan S.M. (2020). Fusion of the Cas9 endonuclease and the vird2 relaxase facilitates homology-directed repair for precise genome engineering in rice. Commun. Biol..

[110] Mishra R., Joshi R.K., Zhao K. (2020). Base editing in crops: Current advances, limitations and future implications. Plant Biotechnol. J..

[111] Zhu H., Li C., Gao C. (2020). Applications of CRISPR-Cas in agriculture and plant biotechnology. Nat. Rev. Mol. Cell. Biol..

[112] Kang B.C., Yun J.Y., Kim S.T., Shin Y., Ryu J., Choi M., Woo J.W., Kim J.S. (2018). Precision genome engineering through adenine base editing in plants. Nat. Plants.

[113] Komor A.C., Kim Y.B., Packer M.S., Zuris J.A., Liu D.R. (2016). Programmable editing of a target base in genomic DNA without double-stranded DNA cleavage. Nature.

[114] Kuang Y., Li S., Ren B., Yan F., Spetz C., Li X., Zhou X., Zhou H. (2020). Base-editing-mediated artificial evolution of *OsALS1 in planta* to develop novel herbicide-tolerant rice germplasms. Mol. Plant.

[115] Li Y., Zhu J., Wu H., Liu C., Huang C., Lan J., Zhao Y., Xie C. (2020). Precise base editing of non-allelic acetolactate synthase genes confers sulfonylurea herbicide resistance in maize. Crop J..

[116] Cheng H., Hao M., Ding B., Mei D., Wang W., Wang H., Zhou R., Liu J., Li C., Hu Q. (2021). Base editing with high efficiency in allotetraploid oilseed rape by A3A-PBE system. Plant Biotechnol. J..

[117] Veillet F., Chauvin L., Kermarrec M.P., Sevestre F., Merrer M., Terret Z., Szydlowski N., Devaux P., Gallois J.L., Chauvin J.E. (2019). The *Solanum tuberosum GBSSI* gene: A target for assessing gene and base editing in tetraploid potato. Plant Cell Rep..

[118] Bharat S.S., Li S., Li J., Yan L., Xia L. (2020). Base editing in plants: Current status and challenges. Crop J..

[119] Park S.-C., Park S., Jeong Y.J., Lee S.B., Pyun J.W., Kim S., Kim T.H., Kim S.W., Jeong J.C., Kim C.Y. (2019). DNA-free mutagenesis of *GIGANTEA* in *Brassica oleracea* var. capitata using CRISPR/Cas9 ribonucleoprotein complexes. Plant Biotechnol. Rep..

[120] Liang Z., Chen K., Li T., Zhang Y., Wang Y., Zhao Q., Liu J., Zhang H., Liu C., Ran Y. (2017). Efficient DNA-free genome editing of bread wheat using CRISPR/Cas9 ribonucleoprotein complexes. Nat. Commun..

[121] Murovec J., Gucek K., Bohanec B., Avbelj M., Jerala R. (2018). DNA-free genome editing of *Brassica oleracea* and *B. rapa* protoplasts using CRISPR-Cas9 ribonucleoprotein complexes. Front. Plant Sci..

[122] Xu J., Hua K., Lang Z. (2019). Genome editing for horticultural crop improvement. Hortic. Res..

[123] Cong L., Ran F.A., Cox D., Lin S., Barretto R., Habib N., Hsu P.D., Wu X., Jiang W., Marraffini L.A. (2013). Multiplex genome engineering using CRISPR/Cas systems. Science.

[124] Mali P., Yang L., Esvelt K.M., Aach J., Guell M., DiCarlo J.E., Norville J.E., Church G.M. (2013). RNA-guided human genome engineering via Cas9. Science.

[125] Guo M., Chen H., Dong S., Zhang Z., Luo H. (2022). CRISPR-Cas gene editing technology and its application prospect in medicinal plants. Chin. Med..

[126] Jiang W., Zhou H., Bi H., Fromm M., Yang B., Weeks D.P. (2013). Demonstration of CRISPR/Cas9/sgRNA-mediated targeted gene modification in Arabidopsis, tobacco, sorghum and rice. Nucleic Acids Res..

[127] Nekrasov V., Staskawicz B., Weigel D., Jones J.D., Kamoun S. (2013). Targeted mutagenesis in the model plant *Nicotiana benthamiana* using Cas9 RNA-guided endonuclease. Nat. Biotechnol..

[128] Svitashev S., Young J.K., Schwartz C., Gao H., Falco S.C., Cigan A.M. (2015). Targeted mutagenesis, precise gene editing, and site-specific gene insertion in maize using Cas9 and guide RNA. Plant Physiol..

[129] Čermák T., Baltes N.J., Čegan R., Zhang Y., Voytas D.F. (2015). High-frequency, precise modification of the tomato genome. Genome Biol..

[130] Jacobs T.B., LaFayette P.R., Schmitz R.J., Parrott W.A. (2015). Targeted genome modifications in soybean with CRISPR/Cas9. BMC Biotechnol..

[131] Wang S., Zhang S., Wang W., Xiong X., Meng F., Cui X. (2015). Efficient targeted mutagenesis in potato by the CRISPR/Cas9 system. Plant Cell Rep..

[132] Varkonyi-Gasic E., Wang T., Voogd C., Jeon S., Drummond R.S.M., Gleave A.P., Allan A.C. (2019). Mutagenesis of kiwifruit centroradialis-like genes transforms a climbing woody perennial with long juvenility and axillary flowering into a compact plant with rapid terminal flowering. Plant Biotechnol. J..

[133] Tripathi J.N., Ntui V.O., Ron M., Muiruri S.K., Britt A., Tripathi L. (2019). CRISPR/Cas9 editing of endogenous *banana streak virus* in the B genome of *Musa spp.* Overcomes a major challenge in banana breeding. Commun. Biol..

[134] Fan D., Liu T., Li C., Jiao B., Li S., Hou Y., Luo K. (2015). Efficient CRISPR/Cas9-mediated targeted mutagenesis in populus in the first generation. Sci. Rep..

[135] Li C., Unver T., Zhang B. (2017). A high-efficiency CRISPR/Cas9 system for targeted mutagenesis in cotton (*Gossypium hirsutum* L.). Sci. Rep..

[136] Fan Y., Xin S., Dai X., Yang X., Huang H., Hua Y. (2020). Efficient genome editing of rubber tree (*Hevea brasiliensis*) protoplasts using CRISPR/Cas9 ribonucleoproteins. Ind. Crops Prod..

[137] Yeap W.C., Norkhairunnisa Che Mohd K., Norfadzilah J., Muad M.R., Appleton D.R., Harikrishna K. (2021). An efficient clustered regularly interspaced short palindromic repeat (CRISPR)/CRISPR-associated protein 9 mutagenesis system for oil palm (*Elaeis guineensis*). Front. Plant Sci..

[138] Huang B., Zhuo R., Fan H., Wang Y., Xu J., Jin K., Qiao G. (2022). An efficient genetic transformation and CRISPR/Cas9-based genome editing system for moso bamboo (*Phyllostachys edulis*). Front. Plant Sci..

[139] Kishi-Kaboshi M., Aida R., Sasaki K. (2017). Generation of gene-edited *Chrysanthemum morifolium* using multicopy transgenes as targets and markers. Plant Cell Physiol..

[140] Shibuya K., Watanabe K., Ono M. (2018). CRISPR/Cas9-mediated mutagenesis of the *EPHEMERAL1* locus that regulates petal senescence in Japanese morning glory. Plant Physiol. Biochem..

[141] Yan R., Wang Z., Ren Y., Li H., Liu N., Sun H. (2019). Establishment of efficient genetic transformation systems and application of CRISPR/Cas9 genome editing technology in *Lilium pumilum* DC. Fisch. and *Lilium longiflorum* white heaven. Int. J. Mol. Sci..

[142] Zhang B., Xu X., Huang R., Yang S., Li M., Guo Y. (2021). CRISPR/Cas9-mediated targeted mutation reveals a role for *AN4* rather than *DPL* in regulating venation formation in the corolla tube of *Petunia hybrida*. Hortic. Res..

[143] Tong C.G., Wu F.H., Yuan Y.H., Chen Y.R., Lin C.S. (2020). High-efficiency CRISPR/Cas-based editing of *Phalaenopsis* orchid *MADS* genes. Plant Biotechnol. J..

[144] Tang Y., Li H., Liu C., He Y., Wang H., Zhao T., Xu X., Li J., Yang H., Jiang J. (2022). CRISPR-Cas9-mediated mutagenesis of the *SlSRM1-like* gene leads to abnormal leaf development in tomatoes. BMC Plant Biol..

[145] Krenek P., Chubar E., Vadovic P., Ohnoutkova L., Vlcko T., Bergougnoux V., Capal P., Ovecka M., Samaj J. (2021). CRISPR/Cas9-induced loss-of-function mutation in the barley *Mitogen-activated protein kinase 6* gene causes abnormal embryo development leading to severely reduced grain germination and seedling shootless phenotype. Front. Plant Sci..

[146] Galli M., Martiny E., Imani J., Kumar N., Koch A., Steinbrenner J., Kogel K.H. (2022). CRISPR/SpCas9-mediated double knockout of barley Microrchidia MORC1 and MORC6a reveals their strong involvement in plant immunity, transcriptional gene silencing and plant growth. Plant Biotechnol. J..

[147] Li Y., Lin Z., Yue Y., Zhao H., Fei X., E L., Liu C., Chen S., Lai J., Song W. (2021). Loss-of-function alleles of *ZmPLD3* cause haploid induction in maize. Nat. Plants.

[148] Zhou J., Zhang R., Jia X., Tang X., Guo Y., Yang H., Zheng X., Qian Q., Qi Y., Zhang Y. (2022). CRISPR-Cas9 mediated *OsMIR168a* knockout reveals its pleiotropy in rice. Plant Biotechnol. J..

[149] Le Rhun A., Escalera-Maurer A., Bratovic M., Charpentier E. (2019). CRISPR-Cas in *Streptococcus pyogenes*. RNA Biol..

[150] Li J., Manghwar H., Sun L., Wang P., Wang G., Sheng H., Zhang J., Liu H., Qin L., Rui H. (2019). Whole genome sequencing reveals rare off-target mutations and considerable inherent genetic or/and somaclonal variations in CRISPR/Cas9-edited cotton plants. Plant Biotechnol. J..

[151] Young J., Zastrow-Hayes G., Deschamps S., Svitashev S., Zaremba M., Acharya A., Paulraj S., Peterson-Burch B., Schwartz C., Djukanovic V. (2019). CRISPR-Cas9 editing in maize: Systematic evaluation of off-target activity and its relevance in crop improvement. Sci. Rep..

[152] Jin S., Zong Y., Gao Q., Zhu Z., Wang Y., Qin P., Liang C., Wang D., Qiu J.L., Zhang F. (2019). Cytosine, but not adenine, base editors induce genome-wide off-target mutations in rice. Science.

[153] Jinek M., Chylinski K., Fonfara I., Hauer M., Doudna J.A., Charpentier E. (2012). A programmable dual-RNA-guided DNA endonuclease in adaptive bacterial immunity. Science.

[154] Lino C.A., Harper J.C., Carney J.P., Timlin J.A. (2018). Delivering CRISPR: A review of the challenges and approaches. Drug Deliv..

[155] Ran F.A., Cong L., Yan W.X., Scott D.A., Gootenberg J.S., Kriz A.J., Zetsche B., Shalem O., Wu X., Makarova K.S. (2015). *In vivo* genome editing using *Staphylococcus aureus* Cas9. Nature.

[156] Schiml S., Fauser F., Puchta H. (2014). The CRISPR/Cas system can be used as nuclease for in planta gene targeting and as paired nickases for directed mutagenesis in Arabidopsis resulting in heritable progeny. Plant J..

[157] Walton R.T., Christie K.A., Whittaker M.N., Kleinstiver B.P. (2020). Unconstrained genome targeting with near-pamless engineered CRISPR-Cas9 variants. Science.

[158] Ren B., Liu L., Li S., Kuang Y., Wang J., Zhang D., Zhou X., Lin H., Zhou H. (2019). Cas9-NG greatly expands the targeting scope of the genome-editing toolkit by recognizing NG and other atypical PAMs in rice. Mol. Plant.

[159] Ge Z., Zheng L., Zhao Y., Jiang J., Zhang E.J., Liu T., Gu H., Qu L.J. (2019). Engineered xCas9 and SpCas9-NG variants broaden PAM recognition sites to generate mutations in Arabidopsis plants. Plant Biotechnol. J..

[160] Li J., Luo J., Xu M., Li S., Zhang J., Li H., Yan L., Zhao Y., Xia L. (2019). Plant genome editing using xCas9 with expanded PAM compatibility. J. Genet. Genom..

[161] Sretenovic S., Yin D., Levav A., Selengut J.D., Mount S.M., Qi Y. (2021). Expanding plant genome-editing scope by an engineered iSpyMacCas9 system that targets A-rich PAM sequences. Plant Commun..

[162] Ha D.I., Lee J.M., Lee N.E., Kim D., Ko J.H., Kim Y.S. (2020). Highly efficient and safe genome editing by CRISPR-Cas12a using CRISPR RNA with a ribosyl-2′-o-methylated uridinylate-rich 3′-overhang in mouse zygotes. Exp. Mol. Med..

[163] Wang Y., Wang M., Zheng T., Hou Y., Zhang P., Tang T., Wei J., Du Q. (2020). Specificity profiling of CRISPR system reveals greatly enhanced off-target gene editing. Sci. Rep..

[164] Swarts D.C., van der Oost J., Jinek M. (2017). Structural basis for guide RNA processing and seed-dependent DNA targeting by CRISPR-Cas12a. Mol. Cell.

[165] Zhang Y., Cheng Y., Fang H., Roberts N., Zhang L., Vakulskas C.A., Niedz R.P., Culver J.N., Qi Y. (2022). Highly efficient genome editing in plant protoplasts by ribonucleoprotein delivery of CRISPR-Cas12a nucleases. Front. Genome Ed..

[166] Zhang L., Zuris J.A., Viswanathan R., Edelstein J.N., Turk R., Thommandru B., Rube H.T., Glenn S.E., Collingwood M.A., Bode N.M. (2021). AsCas12a ultra nuclease facilitates the rapid generation of therapeutic cell medicines. Nat. Commun..

[167] Toth E., Varga E., Kulcsar P.I., Kocsis-Jutka V., Krausz S.L., Nyeste A., Welker Z., Huszar K., Ligeti Z., Talas A. (2020). Improved LbCas12a variants with altered PAM specificities further broaden the genome targeting range of Cas12a nucleases. Nucleic Acids Res..

[168] Malzahn A.A., Tang X., Lee K., Ren Q., Sretenovic S., Zhang Y., Chen H., Kang M., Bao Y., Zheng X. (2019). Application of CRISPR-Cas12a temperature sensitivity for improved genome editing in rice, maize, and Arabidopsis. BMC Biol..

[169] Bernabe-Orts J.M., Casas-Rodrigo I., Minguet E.G., Landolfi V., Garcia-Carpintero V., Gianoglio S., Vazquez-Vilar M., Granell A., Orzaez D. (2019). Assessment of Cas12a-mediated gene editing efficiency in plants. Plant Biotechnol. J..

[170] Schindele P., Puchta H. (2020). Engineering CRISPR/LbCas12a for highly efficient, temperature-tolerant plant gene editing. Plant Biotechnol. J..

[171] Wang M., Mao Y., Lu Y., Tao X., Zhu J.K. (2017). Multiplex gene editing in rice using the CRISPR-Cpf1 system. Mol. Plant.

[172] Li S., Li J., He Y., Xu M., Zhang J., Du W., Zhao Y., Xia L. (2019). Precise gene replacement in rice by RNA transcript-templated homologous recombination. Nat. Biotechnol..

[173] Zhong Z., Zhang Y., You Q., Tang X., Ren Q., Liu S., Yang L., Wang Y., Liu X., Liu B. (2018). Plant genome editing using FnCpf1 and LbCpf1 nucleases at redefined and altered PAM sites. Mol. Plant.

[174] Lee K., Zhang Y., Kleinstiver B.P., Guo J.A., Aryee M.J., Miller J., Malzahn A., Zarecor S., Lawrence-Dill C.J., Joung J.K. (2019). Activities and specificities of CRISPR/Cas9 and Cas12a nucleases for targeted mutagenesis in maize. Plant Biotechnol. J..

[175] Kim H., Kim S.T., Ryu J., Kang B.C., Kim J.S., Kim S.G. (2017). CRISPR/Cpf1-mediated DNA-free plant genome editing. Nat. Commun..

[176] Li B., Liang S., Alariqi M., Wang F., Wang G., Wang Q., Xu Z., Yu L., Naeem Zafar M., Sun L. (2021). The application of temperature sensitivity CRISPR/LbCpf1 (LbCas12a) mediated genome editing in allotetraploid cotton (*G. hirsutum*) and creation of nontransgenic, gossypol-free cotton. Plant Biotechnol. J..

[177] Li B., Rui H., Li Y., Wang Q., Alariqi M., Qin L., Sun L., Ding X., Wang F., Zou J. (2019). Robust CRISPR/Cpf1 (Cas12a)-mediated genome editing in allotetraploid cotton (*Gossypium hirsutum*). Plant Biotechnol. J..

[178] Jia H., Orbovic V., Wang N. (2019). CRISPR-LbCas12a-mediated modification of citrus. Plant Biotechnol. J..

[179] Park H.M., Liu H., Wu J., Chong A., Mackley V., Fellmann C., Rao A., Jiang F., Chu H., Murthy N. (2018). Extension of the crRNA enhances Cpf1 gene editing in vitro and in vivo. Nat. Commun..

[180] Begemann M.B., Gray B.N., January E., Gordon G.C., He Y., Liu H., Wu X., Brutnell T.P., Mockler T.C., Oufattole M. (2017). Precise insertion and guided editing of higher plant genomes using Cpf1 CRISPR nucleases. Sci. Rep..

[181] Li S., Zhang Y., Xia L., Qi Y. (2020). CRISPR-Cas12a enables efficient biallelic gene targeting in rice. Plant Biotechnol. J..

[182] Li S., Li J., Zhang J., Du W., Fu J., Sutar S., Zhao Y., Xia L. (2018). Synthesis-dependent repair of Cpf1-induced double strand DNA breaks enables targeted gene replacement in rice. J. Exp. Bot..

[183] Wolter F., Puchta H. (2019). In planta gene targeting can be enhanced by the use of CRISPR/Cas12a. Plant J..

[184] Kleinstiver B.P., Sousa A.A., Walton R.T., Tak Y.E., Hsu J.Y., Clement K., Welch M.M., Horng J.E., Malagon-Lopez J., Scarfo I. (2019). Engineered CRISPR-Cas12a variants with increased activities and improved targeting ranges for gene, epigenetic and base editing. Nat. Biotechnol..

[185] Li X., Wang Y., Liu Y., Yang B., Wang X., Wei J., Lu Z., Zhang Y., Wu J., Huang X. (2018). Base editing with a Cpf1-cytidine deaminase fusion. Nat. Biotechnol..

[186] Tang X., Lowder L.G., Zhang T., Malzahn A.A., Zheng X., Voytas D.F., Zhong Z., Chen Y., Ren Q., Li Q. (2017). A CRISPR-Cpf1 system for efficient genome editing and transcriptional repression in plants. Nat. Plants.

[187] Tak Y.E., Kleinstiver B.P., Nunez J.K., Hsu J.Y., Horng J.E., Gong J., Weissman J.S., Joung J.K. (2017). Inducible and multiplex gene regulation using CRISPR-Cpf1-based transcription factors. Nat. Methods.

[188] Breinig M., Schweitzer A.Y., Herianto A.M., Revia S., Schaefer L., Wendler L., Cobos Galvez A., Tschaharganeh D.F. (2019). Multiplexed orthogonal genome editing and transcriptional activation by Cas12a. Nat. Methods.

[189] Campa C.C., Weisbach N.R., Santinha A.J., Incarnato D., Platt R.J. (2019). Multiplexed genome engineering by Cas12a and CRISPR arrays encoded on single transcripts. Nat. Methods.

[190] Abudayyeh O.O., Gootenberg J.S., Essletzbichler P., Han S., Joung J., Belanto J.J., Verdine V., Cox D.B.T., Kellner M.J., Regev A. (2017). RNA targeting with CRISPR-cas13. Nature.

[191] Mahas A., Aman R., Mahfouz M. (2019). CRISPR-cas13d mediates robust RNA virus interference in plants. Genome Biol..

[192] Li J., Chen Z., Chen F., Xie G., Ling Y., Peng Y., Lin Y., Luo N., Chiang C.-M., Wang H. (2020). Targeted mrna demethylation using an engineered dcas13b-alkbh5 fusion protein. Nucleic Acids Res..

[193] Abudayyeh O.O., Gootenberg J.S., Franklin B., Koob J., Kellner M.J., Ladha A., Joung J., Kirchgatterer P., Cox D.B.T., Zhang F. (2019). A cytosine deaminase for programmable single-base RNA editing. Science.

[194] Zhu G., Zhu H. (2022). Modified gene editing systems: Diverse bioengineering tools and crop improvement. Front. Plant Sci..

[195] Laforest L.C., Nadakuduti S.S. (2022). Advances in delivery mechanisms of CRISPR gene-editing reagents in plants. Front. Genome Ed..

[196] Maher M.F., Nasti R.A., Vollbrecht M., Starker C.G., Clark M.D., Voytas D.F. (2020). Plant gene editing through de novo induction of meristems. Nat. Biotechnol..

[197] Xu C.L., Ruan M.Z.C., Mahajan V.B., Tsang S.H. (2019). Viral delivery systems for CRISPR. Viruses.

[198] Lau C.H., Suh Y. (2017). In vivo genome editing in animals using aav-CRISPR system: Applications to translational research of human disease. F1000Research.

[199] Kantor B., Bailey R.M., Wimberly K., Kalburgi S.N., Gray S.J. (2014). Methods for gene transfer to the central nervous system. Adv. Genet..

[200] Pausch P., Al-Shayeb B., Bisom-Rapp E., Tsuchida C.A., Li Z., Cress B.F., Knott G.J., Jacobsen S.E., Banfield J.F., Doudna J.A. (2020). CRISPR-casphi from huge phages is a hypercompact genome editor. Science.

[201] Kujur S., Senthil-Kumar M., Kumar R. (2021). Plant viral vectors: Expanding the possibilities of precise gene editing in plant genomes. Plant Cell Rep..

[202] Zhang Y., Zhang Q., Chen Q.J. (2020). Agrobacterium-mediated delivery of CRISPR/Cas reagents for genome editing in plants enters an era of ternary vector systems. Sci. China Life Sci..

[203] Zhang Y., Iaffaldano B., Qi Y. (2021). CRISPR ribonucleoprotein-mediated genetic engineering in plants. Plant Commun..

[204] Li C., Brant E., Budak H., Zhang B. (2021). CRISPR/Cas: A nobel prize award-winning precise genome editing technology for gene therapy and crop improvement. J. Zhejiang Univ. Sci. B.

[205] Zafar K., Sedeek K.E.M., Rao G.S., Khan M.Z., Amin I., Kamel R., Mukhtar Z., Zafar M., Mansoor S., Mahfouz M.M. (2020). Genome editing technologies for rice improvement: Progress, prospects, and safety concerns. Front. Genome Ed..

[206] Ahmad S., Tang L., Shahzad R., Mawia A.M., Rao G.S., Jamil S., Wei C., Sheng Z., Shao G., Wei X. (2021). CRISPR-based crop improvements: A way forward to achieve zero hunger. J. Agric. Food Chem..

[207] Turnbull C., Lillemo M., Hvoslef-Eide T.A.K. (2021). Global regulation of genetically modified crops amid the gene edited crop boom*—*A review. Front. Plant Sci..

[208] Wang T., Zhang H., Zhu H. (2019). CRISPR technology is revolutionizing the improvement of tomato and other fruit crops. Hortic. Res..

[209] van de Wiel C.C.M., Schaart J.G., Lotz L.A.P., Smulders M.J.M. (2017). New traits in crops produced by genome editing techniques based on deletions. Plant Biotechnol. Rep..

[210] Krasileva K.V., Vasquez-Gross H.A., Howell T., Bailey P., Paraiso F., Clissold L., Simmonds J., Ramirez-Gonzalez R.H., Wang X., Borrill P. (2017). Uncovering hidden variation in polyploid wheat. Proc. Natl. Acad. Sci. USA.

[211] Ahmar S., Gill R.A., Jung K.H., Faheem A., Qasim M.U., Mubeen M., Zhou W. (2020). Conventional and molecular techniques from simple breeding to speed breeding in crop plants: Recent advances and future outlook. Int. J. Mol. Sci..

[212] Chen K., Wang Y., Zhang R., Zhang H., Gao C. (2019). CRISPR/Cas genome editing and precision plant breeding in agriculture. Annu. Rev. Plant Biol..

[213] The European Court of Justice (ECJ) Judgement of the Court in Case C-528/16: Court of Justice of the European Union. http://curia.europa.eu/juris/document/document.jsf?text=&docid=204387&pageIndex=0&doclang=EN&mode=lst&dir=&occ=first&part=1&cid=138460.

[214] Waltz E. (2018). With a free pass, CRISPR-edited plants reach market in record time. Nat. Biotechnol..

[215] Waltz E. (2015). A face-lift for biotech rules begins. Nat. Biotechnol..

[216] The Food and Agriculture Organization (FAO) Principles for the Risk Analysis of Foods Derived from Modern Biotechnology. https://www.fao.org/fao-who-codexalimentarius/sh-proxy/fr/?lnk=1&url=https%253A%252F%252Fworkspace.fao.org%252Fsites%252Fcodex%252FStandards%252FCXG%2B44-2003%252FCXG_044e.pdf.

[217] The International Service for the Acquisition of Agri-biotech Applications (ISAAA) Pocket K No. 56: Substantial Equivalence of GM and Non-GM Crops. https://www.isaaa.org/resources/publications/pocketk/56/default.asp#:~:text=In%20other%20words%2C%20substantial%20equivalence,or%20removed%20through%20genetic%20engineering.

[218] United Nations (UN) Parties to the Cartagena Protocol and Its Supplementary Protocol on Liability and Redress. https://bch.cbd.int/protocol/parties/.

[219] European Commission (EC) GMO Legislation. https://ec.europa.eu/food/plants/genetically-modified-organisms/gmo-legislation_en.

[220] Bayer (2021). Annual Monitoring Report on the Cultivation of Mon 810 in 2020.

[221] Belder T.D. (2021). Biotechnology and Other New Production Technologies Annual.

[222] The International Service for the Acquisition of Agri-biotech Applications (ISAAA) Pocket K No. 42: Stacked Traits in Biotech Crops. https://www.isaaa.org/resources/publications/pocketk/42/default.asp.

[223] Vesprini F., Whelan A.I., Goberna M.F., Murrone M.L., Barros G.E., Frankow A., Godoy P., Lewi D.M. (2021). Update of argentina’s regulatory policies on the environmental risk assessment. Front. Bioeng. Biotechnol..

[224] Taverniers I., Papazova N., Bertheau Y., De Loose M., Holst-Jensen A. (2008). Gene stacking in transgenic plants: Towards compliance between definitions, terminology, and detection within the EU regulatory framework. Environ. Biosaf. Res..

[225] Sato S. (2021). Agricultural Biotechnology Annual.

[226] Ahmad A., Munawar N., Khan Z., Qusmani A.T., Khan S.H., Jamil A., Ashraf S., Ghouri M.Z., Aslam S., Mubarik M.S. (2021). An outlook on global regulatory landscape for genome-edited crops. Int. J. Mol. Sci..

[227] European Food Safety Authority (EFSA) (2020). Applicability of the efsa opinion on site-directed nucleases type 3 for the safety assessment of plants developed using site-directed nucleases type 1 and 2 and oligonucleotide-directed mutagenesis. EFSA J..

[228] Strauss S.H., Sax J.K. (2016). Ending event-based regulation of GMO crops. Nat. Biotechnol..

[229] Eckerstorfer M.F., Engelhard M., Heissenberger A., Simon S., Teichmann H. (2019). Plants developed by new genetic modification techniques-comparison of existing regulatory frameworks in the EU and non-EU countries. Front. Bioeng. Biotechnol..

[230] Grassini P., Eskridge K.M., Cassman K.G. (2013). Distinguishing between yield advances and yield plateaus in historical crop production trends. Nat. Commun..

[231] Alexandratos N., Bruinsma J. (2012). World Agriculture Towards 2030/2050: The 2012 Revision.

[232] Brookes G., Barfoot P. (2020). GM crop technology use 1996-2018: Farm income and production impacts. GM Crops Food.

[233] Klumper W., Qaim M. (2014). A meta-analysis of the impacts of genetically modified crops. PLoS ONE.

[234] Li M., Xu J., Gao Z., Tian H., Gao Y., Kariman K. (2020). Genetically modified crops are superior in their nitrogen use efficiency-a meta-analysis of three major cereals. Sci. Rep..

[235] Beatty P.H., Shrawat A.K., Carroll R.T., Zhu T., Good A.G. (2009). Transcriptome analysis of nitrogen-efficient rice over-expressing alanine aminotransferase. Plant Biotechnol. J..

[236] Good A.G., Johnson S.J., De Pauw M., Carroll R.T., Savidov N., Vidmar J., Lu Z., Taylor G., Stroeher V. (2007). Engineering nitrogen use efficiency with alanine aminotransferase. Canad. J. Bot..

[237] Pellegrino E., Bedini S., Nuti M., Ercoli L. (2018). Impact of genetically engineered maize on agronomic, environmental and toxicological traits: A meta-analysis of 21 years of field data. Sci. Rep..

[238] Qaim M. (2020). Bt cotton, yields and farmers’ benefits. Nat. Plants.

[239] Alvarez F., Manalo A., Clarete R. (2021). Economic assessment of GM corn use in the Philippines. Int. J. Food Sci. Agric..

[240] Sexton S.E., Zilberman D., Zivin J.S.G., Perloff J.M. (2011). Land for food and fuel production: The role of agricultural biotechnology. The Intended and Unintended Effects of U.S. Agricultural and Biotechnology Policies.

[241] Brookes G., Barfoot P. (2020). Environmental impacts of genetically modified (GM) crop use 1996–2018: Impacts on pesticide use and carbon emissions. GM Crops Food.

[242] Savary S., Willocquet L., Pethybridge S.J., Esker P., McRoberts N., Nelson A. (2019). The global burden of pathogens and pests on major food crops. Nat. Ecol. Evol..

[243] van Esse H.P., Reuber T.L., van der Does D. (2020). Genetic modification to improve disease resistance in crops. New Phytol..

[244] Ferreira S.A., Pitz K.Y., Manshardt R., Zee F., Fitch M., Gonsalves D. (2002). Virus coat protein transgenic papaya provides practical control of *Papaya ringspot virus* in Hawaii. Plant Dis..

[245] Gonsalves C., Lee D., Gonsalves D. Transgenic virus-resistant papaya: The Hawaiian ’rainbow’ was rapidly adopted by farmers and is of major importance in Hawaii today. APSnet Feature Artic..

[246] Kasote D., Sreenivasulu N., Acuin C., Regina A. (2021). Enhancing health benefits of milled rice: Current status and future perspectives. Crit. Rev. Food Sci. Nutr..

[247] FAO, UNICEF, WFP, WHO (2021). The State of Food Security and Nutrition in the World 2021: Transforming Food Systems for Food Security, Improved Nutrition and Affordable Healthy Diets for All.

[248] De Steur H., Mehta S., Gellynck X., Finkelstein J.L. (2017). GM biofortified crops: Potential effects on targeting the micronutrient intake gap in human populations. Curr. Opin. Biotechnol..

[249] Zhu Q., Wang B., Tan J., Liu T., Li L., Liu Y.G. (2020). Plant synthetic metabolic engineering for enhancing crop nutritional quality. Plant Commun..

[250] Mallikarjuna Swamy B.P., Marundan S., Samia M., Ordonio R.L., Rebong D.B., Miranda R., Alibuyog A., Rebong A.T., Tabil M.A., Suralta R.R. (2021). Development and characterization of GR2E Golden rice introgression lines. Sci. Rep..

[251] World Health Organization (WHO) (2009). Global Prevalence of Vitamin a Deficiency in Populations at Risk 1995–2005: WHO Global Database on Vitamin a Deficiency.

[252] Garg M., Sharma N., Sharma S., Kapoor P., Kumar A., Chunduri V., Arora P. (2018). Biofortified crops generated by breeding, agronomy, and transgenic approaches are improving lives of millions of people around the world. Front. Nutr..

[253] Naqvi S., Zhu C., Farre G., Ramessar K., Bassie L., Breitenbach J., Perez Conesa D., Ros G., Sandmann G., Capell T. (2009). Transgenic multivitamin corn through biofortification of endosperm with three vitamins representing three distinct metabolic pathways. Proc. Natl. Acad. Sci. USA.

[254] Singh S.P., Gruissem W., Bhullar N.K. (2017). Single genetic locus improvement of iron, zinc and beta-carotene content in rice grains. Sci. Rep..

[255] Blancquaert D., Van Daele J., Strobbe S., Kiekens F., Storozhenko S., De Steur H., Gellynck X., Lambert W., Stove C., Van Der Straeten D. (2015). Improving folate (vitamin B9) stability in biofortified rice through metabolic engineering. Nat. Biotechnol..

[256] Storozhenko S., De Brouwer V., Volckaert M., Navarrete O., Blancquaert D., Zhang G.F., Lambert W., Van Der Straeten D. (2007). Folate fortification of rice by metabolic engineering. Nat. Biotechnol..

[257] World Health Organization (WHO) Immunization Coverage. https://www.who.int/news-room/fact-sheets/detail/immunization-coverage.

[258] Kurup V.M., Thomas J. (2020). Edible vaccines: Promises and challenges. Mol. Biotechnol..

[259] Rukavtsova E.B., Rudenko N.V., Puchko E.N., Zakharchenko N.S., Buryanov Y.I. (2015). Study of the immunogenicity of hepatitis B surface antigen synthesized in transgenic potato plants with increased biosafety. J. Biotechnol..

[260] Kumar G.B., Ganapathi T.R., Revathi C.J., Srinivas L., Bapat V.A. (2005). Expression of hepatitis B surface antigen in transgenic banana plants. Planta.

[261] Davod J., Fatemeh D.N., Honari H., Hosseini R. (2018). Constructing and transient expression of a gene cassette containing edible vaccine elements and shigellosis, anthrax and cholera recombinant antigens in tomato. Mol. Biol. Rep..

[262] Karasev A.V., Foulke S., Wellens C., Rich A., Shon K.J., Zwierzynski I., Hone D., Koprowski H., Reitz M. (2005). Plant based HIV-1 vaccine candidate: Tat protein produced in spinach. Vaccine.

[263] Zhang H., Liu M., Li Y., Zhao Y., He H., Yang G., Zheng C. (2010). Oral immunogenicity and protective efficacy in mice of a carrot-derived vaccine candidate expressing UreB subunit against *Helicobacter pylori*. Protein Expr. Purif..

[264] Lossl A.G., Waheed M.T. (2011). Chloroplast-derived vaccines against human diseases: Achievements, challenges and scopes. Plant Biotechnol. J..

[265] Ali Q., Yu C., Hussain A., Ali M., Ahmar S., Sohail M.A., Riaz M., Ashraf M.F., Abdalmegeed D., Wang X. (2022). Genome engineering technology for durable disease resistance: Recent progress and future outlooks for sustainable agriculture. Front. Plant Sci..

[266] Bucchini L., Goldman L.R. (2002). Starlink corn: A risk analysis. Environ. Health Perspect..

[267] Bruhn C.M. Starlink Corn: What Happened. https://ccr.ucdavis.edu/biotechnology/starlink-corn-what-happened.

[268] Seegerweiss Starlink Genetically Modified Corn Contamination. https://www.seegerweiss.com/commercial-litigation/star-link-genetically-modified-corn-seed-settlement/.

[269] Centers for Disease Control and Prevention (CDC) (2001). Investigation of Human Health Effects Associated with Potential Exposure to Genetically Modified Corn.

[270] Ladics G.S., Bartholomaeus A., Bregitzer P., Doerrer N.G., Gray A., Holzhauser T., Jordan M., Keese P., Kok E., Macdonald P. (2015). Genetic basis and detection of unintended effects in genetically modified crop plants. Transgenic Res..

[271] Gu X., Liu L., Zhang H. (2021). Transgene-free genome editing in plants. Front. Genome Ed..

[272] Lu H.P., Liu S.M., Xu S.L., Chen W.Y., Zhou X., Tan Y.Y., Huang J.Z., Shu Q.Y. (2017). CRISPR-s: An active interference element for a rapid and inexpensive selection of genome-edited, transgene-free rice plants. Plant Biotechnol. J..

[273] Aliaga-Franco N., Zhang C., Presa S., Srivastava A.K., Granell A., Alabadi D., Sadanandom A., Blazquez M.A., Minguet E.G. (2019). Identification of transgene-free CRISPR-edited plants of rice, tomato, and Arabidopsis by monitoring dsred fluorescence in dry seeds. Front. Plant Sci..

[274] Gao X., Chen J., Dai X., Zhang D., Zhao Y. (2016). An effective strategy for reliably isolating heritable and Cas9-free Arabidopsis mutants generated by CRISPR/Cas9-mediated genome editing. Plant Physiol..

[275] He Y., Zhu M., Wang L., Wu J., Wang Q., Wang R., Zhao Y. (2018). Programmed self-elimination of the CRISPR/Cas9 construct greatly accelerates the isolation of edited and transgene-free rice plants. Mol. Plant.

[276] The Food and Agriculture Organization (FAO) Women-Users, Preservers and Managers of Agro-Biodiversity. https://www.fao.org/3/x0171e/x0171e03.htm.

[277] Buiatti M., Christou P., Pastore G. (2013). The application of GMOs in agriculture and in food production for a better nutrition: Two different scientific points of view. Genes Nutr..

[278] Lin B.B. (2011). Resilience in agriculture through crop diversification: Adaptive management for environmental change. BioScience.

[279] Rubio L., Galipienso L., Ferriol I. (2020). Detection of plant viruses and disease management: Relevance of genetic diversity and evolution. Front. Plant Sci..

[280] Plumer B. Why Bayer’s Massive Deal to Buy Monsanto Is so Worrisome. https://www.vox.com/2016/9/14/12916344/monsanto-bayer-merger.

[281] Bratspies R. (2017). Owning all the seeds: Consolidation and control in agbiotech. J. Environ. Law.

[282] Demont M., Dillen K., Mathijs E., Tollens E. (2007). GM crops in europe: How much value and for whom?. EuroChoices.

